# Adaptation and validation of two autism-related measures of skills and quality of life in Ethiopia

**DOI:** 10.1177/13623613211050751

**Published:** 2021-12-07

**Authors:** Anton Borissov, Ioannis Bakolis, Bethlehem Tekola, Mersha Kinfe, Caterina Ceccarelli, Fikirte Girma, Rehana Abdurahman, Tigist Zerihun, Charlotte Hanlon, Rosa A Hoekstra

**Affiliations:** 1 King’s College London, UK; 2 Addis Ababa University, Ethiopia; 3 Yekatit 12 Hospital and Medical College, Ethiopia; 4 St. Paul’s Hospital Millennium Medical College, Ethiopia

**Keywords:** autism spectrum disorders, Africa, behavioural measurement, family functioning and support, neurodevelopmental disorders, psychometrics, validation

## Abstract

Although most children with autism and other neurodevelopmental disorders live in low- and middle-income countries, assessment tools are lacking in these settings. This study aims to culturally adapt and validate two questionnaires for use in Ethiopia: the Autism Treatment Evaluation Checklist and the Pediatric Quality of Life Inventory™ Family Impact Module. Both questionnaires were adapted to be suitable for administration in low-literate caregivers and translated using the backward translation procedure. The factor structure, reliability and validity were investigated using caregiver-reports on 300 children with neurodevelopmental disorders or physical health conditions. Confirmatory factor analysis of the Pediatric Quality of Life Inventory™ Family Impact Module data indicated an acceptable fit of the hypothesised eight-factor structure. Internal consistency was high for both measures. Test–retest reliability was excellent for the Autism Treatment Evaluation Checklist and moderate to excellent for the Pediatric Quality of Life Inventory™ Family Impact Module. Both questionnaires demonstrated adequate known-group validity, with moderate to very large effect size group differences between case and control groups. The questionnaires correlated moderately with each other. In conclusion, the Ethiopian adaptations of the Autism Treatment Evaluation Checklist and the Pediatric Quality of Life Inventory™ Family Impact Module are valid and reliable tools for use in parents of children with neurodevelopmental disorders including autism. These adapted measures may also be valuable for use in other low-income settings.

**Lay abstract:**

Although most children with autism and other neurodevelopmental disorders live in low- and middle-income countries, reliable tools to assess these conditions are often not available in these settings. In this study, we adapted two questionnaires developed in Western high-income contexts for use in Ethiopia – the Autism Treatment Evaluation Checklist and the Pediatric Quality of Life Inventory™ Family Impact Module. Both measures are completed by a child’s caregiver and both are relatively short and easy to complete. The Autism Treatment Evaluation Checklist is used to monitor the developmental issues of the child, while the Pediatric Quality of Life Inventory™ Family Impact Module measures the impact of the child’s condition on the caregiver. We translated both tools into the Ethiopian language Amharic, and adapted them to the local cultural context. Three hundred caregivers, half of whom were parents of children with neurodevelopmental disorders, and half were parents of children with physical health problems, completed the questionnaires through a face-to face interview, so that non-literate caregivers could also take part. Both tools performed adequately, measured what we aimed to measure and were reliable. Both the Autism Treatment Evaluation Checklist and Pediatric Quality of Life Inventory™ are suitable tools to assess children with developmental and other health problems in Ethiopia and their caregivers. We believe that more similar tools should be developed or adapted for use in low-income countries like Ethiopia, to gain a better understanding of developmental problems in those settings, and allowing clinicians and service providers to use these tools in their practice. Moreover, these tools can be used in future studies to evaluate interventions to improve support for families.

## Introduction

### Background

Around 95% of all young children with autism spectrum disorder (ASD) or other developmental disabilities live in low- and middle-income countries (LMICs; Olusanya et al., 2018). However, diagnostic and intervention services in these settings are severely lacking (Durkin et al., 2015; Tekola et al., 2016) and public awareness is low (Ruparelia et al., 2016). Moreover, very little is known about autism in these contexts, with large knowledge gaps in many areas of research, such as prevalence and risk factors (Abubakar et al., 2016), the influence of culture and context on identification and diagnosis (de Leeuw et al., 2020) and what types of interventions may work best in low-resource settings (Franz et al., 2017).

One major hurdle is the lack of accessible tools for assessing and monitoring ASD and instruments to use as intervention outcome measures (Ruparelia et al., 2016). Almost all existing tools are developed in high-income Western contexts, and are often evaluated in families predominantly of high socio-economic class, who are highly educated, urban and white (de Vries, 2016; Durkin et al., 2015). In low-resource contexts, tools need to be accessible to people with various literacy levels and from different socio-economic groups (de Vries, 2016). Another barrier is that many established instruments have an expensive and often time-limited licence (Durkin et al., 2015). Some tools are lengthy and require administration by a trained professional, making them unsuitable for wide use in LMIC settings. While there are clear guidelines for best practice in cultural adaptation of tools (Kirmayer & Swartz, 2014), these are not always implemented in adaptation of autism tools (Al Maskari et al., 2018; although see, for example, Smith et al. (2017) for an example of a carefully executed adaptation).

This study was conducted in Ethiopia, a low-income country in sub-Saharan Africa. Two established tools that are caregiver-reported and relatively quick to complete were employed. First, the Autism Treatment Evaluation Checklist (ATEC; Rimland & Edelson, 1999), a questionnaire assessing a range of developmental skills and behaviours relevant to autism. The ATEC was designed to be used in intervention studies to evaluate the intervention’s impact, and has also been used to monitor the trajectory of the condition (Magiati et al., 2011; Mahapatra et al., 2018). The ATEC was selected over other measures of autism-related symptoms because it is freely available, has been previously translated into many different languages, can be used a measure of change and assesses broad functioning and not exclusively autism-related symptoms. The second tool was the Pediatric Quality of Life Inventory™ (PedsQL™; Varni et al., 1999). Family Impact Module (FIM; Varni et al., 2004) used to assess the impact of the child’s condition on family functioning.

The aim of this study was to adapt and translate the ATEC and the PedsQL™ FIM for use in Ethiopia and to evaluate their psychometric properties. The tools complement each other well: the ATEC measures the child’s symptoms, while the PedsQL™ FIM provides insight into the impact of the child’s condition on the caregiver. Both tools are also multi-purpose: they could be used to monitor the state of child and caregiver or to evaluate the efficacy of an intervention. Furthermore, both tools could be suitable for children with neurodevelopmental disorders and not just ASD. Even though the ATEC was designed with ASD in mind, many of its items assess development more generally (Charman et al., 2004).

This study had several objectives. First, we wanted to assess the reliability of the ATEC and the PedsQL™ FIM, specifically their internal consistency and stability (test–retest reliability). Second, we aimed to demonstrate the known-group validity of the tools. We hypothesised that caregivers of children with neurodevelopmental disorders would score higher on the ATEC than caregivers of children with physical health conditions. We did not have such expectations for the PedsQL™ FIM because we could not find evidence suggesting that caregivers of children with neurodevelopmental disorders would be more severely impacted than caregivers of children with other clinical conditions. Third, we wanted to assess the convergent validity of the ATEC and the PedsQL™ FIM. We hypothesised that they would be associated with higher severity of both ASD and intellectual disability (ID). We also expected that the ATEC and the PedsQL™ FIM would be associated with each other. Fourth, we aimed to confirm the factor structure of the ATEC and the PedsQL™ FIM as evidence of construct validity.

## Materials and methods

### Setting and participants

The study recruited participants at St. Paul’s Millennium Medical College and Yekatit 12, two state-owned hospitals in Addis Ababa, the capital city of Ethiopia. Each hospital has both a pediatrics and a child mental health clinic, the only two child mental health clinics in the country. Eligible participants were families with a child between 2 and 9 years of age that had been formally diagnosed with a health problem. The pediatrics clinic recruited children with physical health conditions (clinical control group), and the mental health clinic recruited children with either neurodevelopmental disorders alone or with a comorbid mental health condition (case group), both in accordance with the *Diagnostic and Statistical Manual of Mental Disorders* (5th ed.; DSM-5; American Psychiatric Association, 2013). No standardised diagnostic tests are available in Ethiopia; therefore, clinicians relied on their clinical judgement based on clinical interviews with the caregiver and observation and interaction with the child for the neurodevelopmental assessments. The clinical diagnoses in the child mental health clinic were provided by general psychiatrists without specialist expertise in child psychiatry, since this specialty training is not available in Ethiopia. Where possible, neurodevelopmental evaluations were conducted over two separate appointments. However, if the family could only come to the clinic once (e.g. because the family lives in a rural area far from the child mental health clinic), all assessments were done within a single day.

Due to limited awareness, high levels of stigma and lack of available resources in Ethiopia, children attending the child mental health clinics tend to have more severe forms of developmental delay, most children are minimally verbal and co-occurring epilepsy is common. Children with autism who are highly verbal and do not have co-occurring ID do not typically come to clinical attention in Ethiopia.

When present at either clinic, eligible families were approached by the attending clinician and handed a flyer containing general information on the study. Study data collection and consent taking was done by nurses at the clinics who worked independently from the clinicians supporting the families. All instruments were administered by a trained data collector through a face-to-face interview. All participants provided written consent or consent through a witness statement. Participation in the study was voluntary, and families were remunerated for their time and travel expenses. From each recruited family, only a single caregiver participated. A subset of participants was then invited to complete the questionnaires again approximately 3 weeks later. Data collection took place between August 2018 and May 2019.

### Instruments

#### ATEC

The ATEC has 77 items across four subscales: Speech/Language/Communication (14 items), Sociability (20 items), Sensory/Cognitive Awareness (18 items) and Health/Physical/Behaviour (25 items). Respondents are asked to indicate to what extent that behaviour or trait holds true for their child. Items of subscales 1–3 are scored on a 3-point Likert-type scale, ranging from 0 to 2. Answer categories are *Not true*, *Somewhat true*, *Very True* for subscale 1, and *Not descriptive*, *Somewhat descriptive*, *Very descriptive* for subscales 2 and 3. Items of subscale 4 are scored on a 4-point Likert-type scale, ranging from 0 to 3 (*Not a problem*, *Moderate Problem*, *Minor Problem* and *Serious Problem*). After reverse coding relevant items, all items are summed to derive an ATEC total score between 0 and 179, with higher score indicating more ASD-related behaviours or developmental delays. The ATEC was originally developed in English but is now available in 22 languages (Rimland & Edelson, 2020b). It is free to use for non-profit purposes. The English version has been found to have very high internal consistency (Magiati et al., 2011; Rimland & Edelson, 2020a). Even though the ATEC was not designed to be used as a screening tool, significant correlations between the ATEC and diagnostic instruments for ASD have been observed (Geier et al., 2013; Magiati et al., 2011), suggesting high convergent validity. The ATEC has also been validated for use in Brazil, Iran and Thailand (Freire et al., 2018; Memari et al., 2013; Sunakarach & Kessomboon, 2018). In Brazil and Iran, high temporal stability was additionally demonstrated.

#### PedsQL™ FIM

The PedsQL™ FIM has two versions: standard and acute. In this study, the acute version was used, assessing impact of the child’s condition over the last 7 days. We used the acute version because our longer-term goal is to use the PedsQL™ FIM as outcome measure in an intervention of relatively short duration (Tekola, Girma, et al., 2020). Moreover, based on prior experience, the Ethiopian clinicians and researchers in our team felt caregivers would find it easier to reflect on the past 7 days rather than a full month. The scale comprises 36 items across 8 subscales: Physical Functioning (6 items), Emotional Functioning (5 items), Social Functioning (4 items), Cognitive Functioning (5 items), Communication (3 items), Worry (5 items), Daily Activities (3 items) and Family Relationships (5 items). Each item describes a specific problem related to functioning, and respondents are asked to indicate how often they have experienced it over the past week. All items are scored on a 5-point Likert-type scale ranging from 0 *Never*, to 4 *Almost always*. To calculate the PedsQL™ FIM total score, all items are reverse-coded and rescaled to 0, 25, 50, 75 and 100. The total score is expressed as the mean item score, ranging between 0 and 100, with a higher score indicating better functioning. There are also two other scores that could be obtained for the PedsQL™ FIM: the Parent health-related quality of life (HRQL) summary score (includes the Physical Functioning, Emotional Functioning, Social Functioning and Cognitive Functioning subscales) and the Family Functioning summary score (includes the Daily Activities and Family Relationship subscales). The Communication and Worry subscales are only used for the total score.

The PedsQL™ FIM was developed in English but is now available in 49 languages (Varni, 2020). The English version has demonstrated excellent internal consistency (Medrano et al., 2013; Panepinto et al., 2009; Varni et al., 2004). There is some evidence to suggest that the PedsQL™ FIM discriminates well between children with different severity of the same condition (Limbers et al., 2011), but may not discriminate well between children with different chronic conditions (Hsieh et al., 2009; Panepinto et al., 2009). The PedsQL™ FIM has been validated in Malaysia, China, Croatia and Brazil (Ab Rahman et al., 2011; Chen et al., 2011; Knez et al., 2015; Scarpelli et al., 2008). In Brazil, high test–retest reliability was also shown. Finally, the hypothesised factor structure of the PedsQL™ FIM has been confirmed for the English version (Medrano et al., 2013). Use of the PedsQL™ FIM incurs a fee for funded research but is free to use in unfunded studies and thus accessible for use by researchers in low-resource contexts.

### Cross-cultural adaptation

The ATEC and the PedsQL™ FIM were adapted for use in Ethiopia with the goal to produce a version of each that was conceptually equivalent (Prince, 2013) to the original. Both instruments were translated into the Ethiopian language Amharic using the backward translation procedure. In the case of the PedsQL™ FIM, two independent translations were done in compliance with guidelines provided by the copyright holder (Varni, 2020). The translated tools were evaluated by a translation consensus committee consisting of four Amharic native speakers fluent in English: two psychiatrists, one research coordinator and one postdoctoral researcher with a background in psychology and anthropology. The draft versions were pre-tested in cognitive interviews with 20 participants, which took place between May and June 2018, to determine if the items and instructions were easily understood, and adjustments were made where necessary. After that, the final translated versions of the ATEC and the PedsQL™ FIM were established.

The most substantial change introduced in the translated versions was that the questionnaires were no longer self-administered but instead administered through a face-to-face interview, so that non-literate caregivers could also participate. Most items were also rephrased into questions, as findings from the cognitive interviews showed that they were more easily understood that way. For example, item 1 on subscale 3 of the ATEC (Sensory/Cognitive Awareness) was changed from ‘Responds to own name’ to ‘Does your child respond to his or her own name?’ Similarly, item 1 on the Family Relationships subscale of the PedsQL™ FIM was changed from ‘Lack of communication between family members’ to ‘Was there lack of communication between family members?’

In the case of the ATEC, only items of subscales 1–3 were rephrased into questions, and answer categories of subscale 1 and 3 were changed from *Not true*/*Not descriptive*, *Somewhat true*/*Somewhat descriptive*, *Very True*/*Very descriptive* to *Yes*, *No* and *Sometimes*. The switched order (first determining if an item applied *Yes*/*No*, and then whether it only *Sometimes* applied) was found to be easier to understand by participants and easier to administer by data collectors in the context of an interview as opposed to self-administration. In addition, answer categories for subscale 2 (Sociability) were changed from *Not descriptive*, *Somewhat descriptive*, *Very descriptive* to *Yes, s/he does*, *No, s/he doesn’t* and *Sometimes*. In some instances, extra words were added to further clarify the answers and to make negatively worded items less ambiguous. For example, item 4 on subscale 2 (‘Is he or she uncooperative and resistant?’) has the following answer categories: *Yes, s/he is resistant*, *No, s/he isn’t resistant* and *Sometimes*. Some items of subscale 2 were also translated in such a way that they indicated a skill rather than a difficulty. For example, item 16 was changed from ‘Lacks friends/companions’ to ‘Does he or she have friends/companions?’. Those items were reverse-coded before calculating the total score of the ATEC. The complete Amharic questionnaires as well as their English equivalents are made available in the Supplementary Material, with the knowledge and permission of the respective copyright holders.

### Demographic and clinical data

Demographic information on the families was also collected (including age, gender, marital status, religion, level of education and occupation), as well as clinician-assigned severity levels of ASD and ID (when applicable and where available). The following severity levels were employed for both conditions: 1: *mild*, 2: *moderate*, 3: *severe*, corresponding with the severity ratings as described in the DSM-5. The severity ratings were provided by general psychiatrists.

### Ethical considerations

The study and its procedure were approved by the Institutional Review Board of the College of Health Sciences of Addis Ababa University (062/16/Psy) and by the Psychiatry, Nursing and Midwifery Research Ethics Subcommittee at King’s College London (reference no. HR-16/17-3489).

### Community involvement

This research benefits from the long-term involvement of a group of around 20 stakeholders in Addis Ababa, comprising parents of children with developmental disorders, directors of special schools for children with developmental disorders and representatives of local and international Non-Governmental Organisations (NGOs) as well as experts in child mental health and education. This project advisory board advises the research team on important research questions, methods and measures. Moreover, we listened to the feedback from the caregivers participating in the cognitive interviews, who especially highlighted that the PedsQL™ FIM measures aspects of great importance to them. This study did not benefit from input of autistic people, because autism in Ethiopia only comes to clinical attention when there is also substantial ID, and therefore practically all our autistic participants were non-verbal.

### Psychometric evaluation of ATEC and the PedsQL™ FIM

#### Data analysis

IBM SPSS Statistics 26.0 (IBM Corp., 2019) was used when conducting data quality and consistency checks to allow for cleaning the data. The cleaned data set was then analysed with R 3.6.2 (R Core Team, 2019). For the first time point and across control and case groups, the quartiles of the distribution of every item were calculated, as well as the mean and standard deviation of all subscale, total and summary scores. Missing values were imputed with the median of the respective item for all participants. To test whether the demographic backgrounds differed between cases and controls, chi-square (*χ*^2^) tests were used for ordinal or dichotomous variables, and *t*-tests for continuous variables.

#### Factor structure

Confirmatory factor analysis (CFA) with the diagonally weighted least squares scale-shifted estimation method (DWLSSS) for ordinal data was employed to assess the hypothesised eight-factor structure of the PedsQL™ FIM. CFA was not run for ATEC as our sample size was too small to evaluate the factor structure of this 77-item measure. The lavaan package (Rosseel, 2012) was used to fit the CFA model. Structural equation modelling (SEM) was implemented, and all latent factors were standardised, allowing factor loadings to be freely estimated. The model was plotted with the semPlot package (Epskamp, 2019). Standardised factor loading coefficients were reported, as well as the following goodness-of-fit statistics: the root mean square error of approximation (RMSEA), the standardised root mean square residual (SRMR), the comparative fit index (CFI) and the Tucker–Lewis index (TLI). The model fit was considered acceptable if the RMSEA was <0.07, the SRMR was <0.08 and both the CFI and the TLI were ⩾0.95 (Hooper et al., 2008). A 90% confidence interval (CI) was adopted for the RMSEA. In order to improve the fit of the model, residual correlations with the largest modification indices (⩾30) were allowed, as long as the corresponding indicators (items) were similarly phrased or appeared to measure the same construct.

#### Reliability

The reliability of the ATEC and the PedsQL™ FIM was considered by assessing internal consistency using Cronbach’s alpha (*α*) for all subscales and for the total and summary scores. A coefficient of ⩾0.7 was considered to be acceptable (Kline, 2000). Coefficients of 0.8−0.89 and ⩾0.9 were interpreted as good and excellent, respectively. Intraclass correlation coefficient (ICC) was employed to assess the strength of agreement for each questionnaire between the first and the second time points. ICC was utilised for all subscales and for the total and summary scores. ICC was calculated with the irr package (Gamer et al., 2019) and values were interpreted as follows: <0.5: poor, 0.5−0.74: moderate, 0.75−0.89: good and ⩾0.9: excellent (Koo & Li, 2016).

#### Validity

The construct validity of a measure is supported by evidence that scores on the instrument are related to other measures, or participant characteristics in a hypothesised manner. Two aspects of construct validity were assessed: (1) known-groups method and (2) convergent validity.

Known-group validity was assessed using multiple linear regression. Demographic variables that significantly differed between groups were included as covariates in the model. The mean difference between the control and case groups, adjusted for the covariates, were calculated for each score, along with a 95% CI. Cohen’s *d* was used to measure the effect size, and was estimated with the effect size package (Ben-Shachar et al., 2020). Guidelines by Cohen (2013) were used to interpret effect sizes: for absolute values, 0.2 was considered small, 0.5 moderate and 0.8 large.

Convergent validity was assessed by (1) exploring the association between the total scores of the questionnaires and severity of ASD and ID; and (2) assessing the correlation between both questionnaires. The Spearman rank correlation coefficient (*ρ*) was used when exploring the association between the tools and ASD/ID severity. Because the same clinician-assigned severity levels were used for both ASD and ID ratings, they were merged into a single variable and the correlation of that variable with the total scores was computed. When merging, if a child had differing severity levels for both ASD and ID, the higher level of the two was selected. To measure the association between the total scores of the ATEC and the PedsQL™ FIM, the Pearson correlation coefficient (*r*) was utilised. Correlation coefficients were calculated with the Hmisc package (Harrell, 2020). Absolute values of coefficients were interpreted as follows: <0.1: no or negligible correlation, 0.1−0.39: weak correlation, 0.4−0.69: moderate correlation, 0.7−0.89: strong correlation and ⩾0.9: very strong correlation (Schober et al., 2018).

## Results

### Data accessibility

The raw study data, including individual item responses for both the ATEC and PedsQL™ FIM are made openly available on the Figshare repository (see accompanying link on the journal’s website).

### Sample characteristics

Three hundred caregivers participated in the study. The case group (*n* = 139) included children with the following neurodevelopmental disorders: ASD, attention deficit hyperactivity disorder (ADHD), ID, language disorder, global developmental delay and Down’s syndrome. A full breakdown of the neurodevelopmental conditions in the sample is included in Table 1. The control group (*n* = 139) included children with a range of different physical health conditions, including severe community-acquired pneumonia, segmental arterial mediolysis, congestive heart failure and asthma (see Table A.1 in the Supplementary Material for a complete list). The remaining participants (rest group; *n* = 22) included children with epilepsy or a mental health condition (major depressive disorder, generalised anxiety disorder, psychotic disorder, bipolar disorder) and therefore did not meet inclusion criteria for either the case or control group. One participant in the control group was removed from the analysis of the PedsQL™ FIM because they had 19 missing values (more than 50%). Five other participants had one missing item each, and two participants had five missing items each. Those participants were retained.

**Table 1.** Neurodevelopmental disorders in the case group (*n* = 139), comorbidities included.

Condition(s)	Count	%
ASD	55	40%
ADHD	28	20%
ID	19	17%
LD	6	4%
DS	1	0.7%
GDD	1	0.7%
ASD + ID	11	8%
ASD + ADHD	6	4%
ASD + LD	2	1%
ASD + GDD	1	0.7%
ADHD + ID	2	1%
ADHD + LD	1	0.7%
ID + DS	2	1%
ID + GDD	1	0.7%
DS + GDD	1	0.7%
ASD + ADHD + ID	1	0.7%
ADHD + ID + DS	1	0.7%
Total	139	100%

ASD: autism spectrum disorder; ADHD: attention deficit hyperactivity disorder; ID: intellectual disability; LD: language disorder; DS: Down’s syndrome; GDD: global developmental delay.

Of the 300 initial participants, 40 returned to complete the questionnaires a second time. Eight participants had their retest scores removed due to having a problematic response pattern (i.e. they indicated a single answer category on all or almost all items). All eight participants were in the control group and attended the same clinic, suggesting that this concerned a quality control issue of the retest data collection. The final retest sample consisted of 32 participants: 12 in the control group, 19 in the case group and one in the rest group. The mean time difference between the first and the second time points was 20.55 days (SD = 13.81).

Demographic characteristics of the sample across groups are presented in Table 2. The age of caregivers did not differ between the case (M = 34.95, SD = 7.66) and control groups (M = 33.4, SD = 6.59), *t*(276) = 1.81, *p* = 0.072. However, children in the case group (M = 5.34, SD = 1.84) were significantly older than children in the control group (M = 4.32, SD = 2), *t*(276) = 4.43, *p* < 0.001. The case group also had significantly more boys than girls compared to the control group, *χ*^2^(1, *N* = 278) = 18.19, *p* < 0.001. Across both groups, around half of the caregivers had no more than primary school level education.

**Table 2.** Descriptive statistics (*N* = 300).

Sample characteristics	Control (*n* = 139)		Case (*n* = 139)		Rest (*n* = 22)	
	*n*	%	*n*	%	*n*	%
*Caregivers* Gender***, *χ*^2^(1, *N* = 278) = 18.56, *p* < 0.001						
Male	43	30.9	14	10.1	7	31.8
Female	96	69.1	125	89.9	15	68.2
Education, *χ*^2^(4, *N* = 278) = 3.05, *p* = 0.549						
No formal education	20	14.4	20	14.4	4	18.2
Primary school	49	35.3	42	30.2	12	54.5
Secondary school	46	33.1	47	33.8	4	18.2
Diploma	12	8.6	11	7.9	1	4.5
College	9	6.5	17	12.2	0	0
Missing	3	2.2	2	1.4	1	4.5
Occupation*, *p* = 0.02 (Fisher’s exact test)						
Farmer	7	5.0	5	3.6	4	18.2
Housewife	41	29.5	72	51.8	13	59.1
Merchant	15	10.8	16	11.5	2	9.1
Student	1	0.7	0	0	0	0
Civil servant	11	7.9	6	4.3	1	4.5
Daily labourer	19	13.7	11	7.9	0	0
Other	8	5.8	4	2.9	0	0
Missing	37	26.6	25	18	2	9.1
Marital status, *p* = 0.363 (Fisher’s exact test)						
Married	122	87.8	111	79.9	18	81.8
Single	4	2.9	5	3.6	1	4.5
Divorced	10	7.2	18	12.9	3	13.6
Widowed	3	2.2	4	2.9	0	0
Missing	0	0	1	0.7	0	0
Area of residence***, *χ*^2^(1, *N* = 278) = 15.06, *p* < 0.001						
Rural	45	32.4	18	12.9	6	27.3
Urban	91	65.5	118	84.9	16	72.7
Missing	3	2.2	3	2.2	0	0
Religion**, *p* = 0.004 (Fisher’s exact test)						
Orthodox Christian	95	68.3	79	56.8	15	68.2
Protestant Christian	18	12.9	11	7.9	1	4.5
Catholic Christian	1	0.7	1	0.7	0	0
Muslim	23	16.5	47	33.8	6	27.3
Other	2	1.4	0	0	0	0
Missing	0	0	1	0.7	0	0
Relationship to the child***, *p* < 0.001 (Fisher’s exact test)						
Mother	94	67.6	116	83.5	14	63.6
Father	41	29.5	13	9.4	6	27.3
Extended family	4	2.9	5	3.6	2	9.1
Other	0	0	3	2.2	0	0
Missing	0	0	2	1.4	0	0
*Children* Gender***, χ^2^(1, N = 278) = 18.19, p < 0.001						
Male	69	49.6	104	74.8	13	59.1
Female	69	49.6	35	25.2	9	40.9
Missing	1	0.7	0	0	0	0

Some characteristics differed significantly between the control and case groups, as assessed by a *χ*^2^ test or Fisher’s exact test.

* *p* < 0.05; **p < 0.01; ***p < 0.001.

### Item statistics

The quartiles of the distribution of every item for the first time point and across control and case groups are available in the Supplementary Material (Tables A.2 and A.3). For the ATEC, responses showed good variation on most items in the case group but not in the control group, indicating a floor effect in the control group. For the PedsQL™ FIM, most items showed a good variation of answers in both groups.

### Factor structure

**Figure 1.**
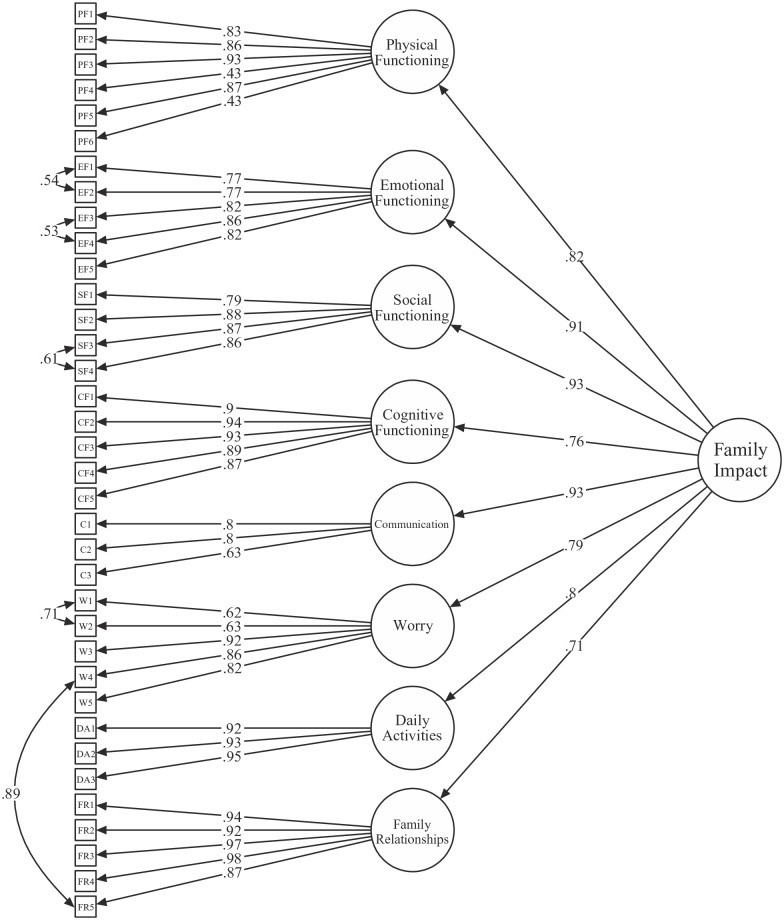
Confirmatory factor analysis of the PedsQL™ FIM. Residual correlations with a modification index of ⩾30 were allowed. All standardised factor loadings and residual correlations were statistically significant (*p* < 0.001).

The CFA conducted to assess the eight-factor structure of the PedsQL™ FIM included all 299 valid observations. All factor loadings were significant and ranged between 0.43 and 0.98. Five residual correlations with a modification index of ⩾30 were allowed between pairs of indicators (items) that had similar phrasing or overlapping concepts. Figure 1 includes a visualisation of the model. The model fit was acceptable with an RMSEA of 0.068 (90% CI = 0.063, 0.073), an SRMR of 0.067, a CFI of 0.975 and a TLI of 0.973.

### Subscale, total and summary scores statistics

The mean and standard deviation of all subscales, total and summary scores across control and case groups are shown in Table 3 (ATEC) and Table 4 (PedsQL™ FIM), along with the respective mean differences and effect sizes. The tables also include Cronbach’s alpha coefficients and ICCs of the scores.

**Table 3.** ATEC: subscale and total scores statistics.

	*N* items	Control	Case	Δa*M* (95% CI)	*d*	Control	Case	ICC (95% CI)
		M (SD)				*α*		
Total score	77	30.6 (26.26)	81.88 (31.58)	49.15*** (40.24, 58.07)	1.30	0.96	0.95	0.96*** (0.93, 0.98)
Speech/language/communication	14	4.41 (6.78)	18.92 (8.16)	13.75*** (11.46, 16.04)	1.42	0.94	0.94	0.98*** (0.95, 0.99)
Sociability	20	8.6 (5.83)	20.12 (9.33)	11.31*** (8.76, 13.87)	1.05	0.76	0.87	0.88*** (0.76, 0.94)
Sensory/cognitive awareness	18	7.82 (6.95)	20.32 (9.91)	13.32*** (10.65, 15.98)	1.18	0.90	0.92	0.94*** (0.89, 0.97)
Health/physical/behaviour	25	9.76 (11.37)	22.53 (12.63)	10.77*** (6.85, 14.69)	0.65	0.91	0.84	0.94*** (0.88, 0.97)

Δa*M*: adjusted mean difference; *d*: Cohen’s *d*, *α*: Cronbach’s alpha.

A higher score indicates more developmental problems in children. Mean differences between control (*n* = 139) and case (*n* = 139) groups were tested for significance and adjusted for gender of caregiver, occupation, area of residence, religion and relationship to the child, as well as gender and age of child. ICCs were calculated for the retest participants (*n* = 32).

*** *p* < 0.001.

**Table 4.** PedsQL™ FIM: subscale, total and summary scores statistics.

	*N* items	Control	Case	Δa*M* (95% CI)	*d*	Control	Case	ICC (95% CI)
		M (SD)				*α*		
Total score	36	71.11 (20.16)	54.14 (17.86)	−12.94*** (−18.71, −7.18)	−0.53	0.97	0.94	0.91*** (0.78, 0.96)
Parent HRQL summary score	20	71.95 (21.35)	55.02 (18.33)	−12.56*** (−18.64, −6.48)	−0.49	0.96	0.91	0.85*** (0.7, 0.92)
Physical functioning	6	69.6 (21.29)	57.31 (20.75)	−8.38* (−14.86, −1.89)	−0.31	0.87	0.80	0.86*** (0.72, 0.93)
Emotional functioning	5	65 (27.05)	43.67 (24.33)	−15.44*** (−23.43, −7.44)	−0.46	0.91	0.83	0.82*** (0.66, 0.91)
Social functioning	4	76.63 (23.47)	49.01 (29.08)	−24.84*** (−33.21, −16.48)	−0.70	0.89	0.86	0.63*** (0.36, 0.8)
Cognitive functioning	5	77.97 (23.55)	68.42 (22.23)	−4.89 (−12.04, 2.25)	−0.16	0.95	0.90	0.86*** (0.73, 0.93)
Communication	3	76.15 (22.85)	63.49 (22.6)	−9.58** (−16.75, −2.4)	−0.32	0.70	0.59	0.78*** (0.6, 0.89)
Worry	5	56.7 (27.73)	35.22 (26.1)	−16.13*** (−24.57, −7.7)	−0.45	0.87	0.80	0.72*** (0.49, 0.85)
Family functioning summary score	8	76.11 (20.94)	60.25 (25.52)	−13.16*** (−20.61, −5.7)	−0.42	0.91	0.89	0.79*** (0.61, 0.89)
Daily activities	3	71.44 (27.94)	53.18 (29.67)	−12.88** (−21.78, −3.82)	−0.34	0.94	0.90	0.68*** (0.44, 0.83)
Family relationships	5	78.91 (21.71)	64.5 (31.86)	−13.37** (−22.59, −4.15)	−0.34	0.91	0.95	0.7*** (0.47, 0.84)

Δa*M*: adjusted mean difference; *d*: Cohen’s *d; α*: Cronbach’s alpha.

A higher score indicates better functioning of caregivers. Mean differences between control (*n* = 138) and case (*n* = 139) groups were tested for significance and adjusted for gender of caregiver, occupation, area of residence, religion and relationship to the child, as well as gender and age of child. ICCs were calculated for the retest participants (*n* = 32).

* *p* < 0.05; **p < 0.01; ***p < 0.001.

### Reliability

#### Internal consistency

Cronbach’s alpha coefficients are available in Table 3 (ATEC) and Table 4 (PedsQL™ FIM). For the total ATEC, internal consistency was excellent (*α* of 0.96 in the control and 0.95 in the case group), and acceptable to excellent for the ATEC subscales (*α* ranging between 0.76 and 0.94). For the total PedsQL™ FIM, coefficients were excellent (*α* of 0.97 in the control and 0.94 in the case group) and good to excellent for the PedsQL™ FIM subscales (*α* ranging between 0.80 and 0.96), with the exception of the Communication subscale where *α* of 0.70 was observed in the control group (marginally acceptable) and 0.59 in the case group (not acceptable). Dropping the third item of the communication subscale would boost *α* in the case group to 0.72.

#### Test–retest reliability

For the total ATEC, ICC was 0.96, indicating excellent agreement between the first and the second time points. ICCs for the subscales ranged between 0.88 and 0.98 (good to excellent). For the total PedsQL™ FIM, ICC was 0.91, indicating excellent agreement. ICCs for the subscales ranged between 0.63 and 0.86 (moderate to good). However, for some subscales (Social Functioning, Worry, Daily Activities and Family Relationships), the lower bound of the confidence interval was in the poor range.

### Validity

#### Known-group validity

For the ATEC, all subscale and total scores were significantly higher for participants in the case group, indicating more developmental problems in children with neurodevelopmental disorders. Effect sizes were large to very large, ranging between 0.65 and 1.42. For the PedsQL™ FIM, all subscale, summary and total scores were lower for participants in the case group, indicating lower functioning (and greater impact of their child’s condition) in caregivers of children with neurodevelopmental disorders compared to children with other health conditions. Differences were significant for all scores, except for the Cognitive Functioning subscale. Effect sizes were small to moderate, ranging between −0.16 and −0.70.

#### Convergent validity

Clinician’s ratings of severity of ASD and ID were available for 70 of the children with ASD and/or ID: 28 *mild*, 34 *moderate* and 8 *severe*. Correlation between severity and the total score of the ATEC was weak and non-significant (*n* = 70, *ρ* = 0.19, *p* = 0.117). Correlation between severity and the total score of the PedsQL™ FIM was weak and non-significant (*n* = 70, *ρ* = −0.18, *p* = 0.142). Correlation between the ATEC and the PedsQL™ FIM was moderate and significant (*n* = 299, *r* *=* −0.59, *p* < 0.001), indicating greater impact of the child’s condition in caregivers who reported more developmental problems in their children.

## Discussion

This study set out to adapt the ATEC and the PedsQL™ FIM for use in Ethiopia and to investigate their psychometric properties in a clinical sample. The case and control groups included caregivers of children with neurodevelopmental disorders and with physical health conditions, respectively. The adapted translated questionnaires demonstrated mostly good internal consistency and test–retest reliability in both groups. The adapted PedsQL™ FIM appeared to have construct validity, with CFA of the PedsQL™ FIM yielding positive and significant factor loadings, and an acceptable model fit according to all four fit indices. Known-group validity was also observed, with children with neurodevelopmental disorders scoring lower on the ATEC than clinical controls, and caregivers of children with neurodevelopmental disorders reporting a greater impact of their child’s condition on their own functioning. In regard to convergent validity, we hypothesised that both questionnaires would correlate with severity of ASD and ID, but the correlations were only modest and non-significant. The ATEC and PedsQL™ FIM correlated moderately and significantly in the expected direction.

For the ATEC, internal consistency was mostly good to excellent, which is in line with previously reported coefficients for the original English version (Magiati et al., 2011; Rimland & Edelson, 2020a) and for the Iranian version (Memari et al., 2013). To our knowledge, this study was the first validation study on the ATEC to employ a clinical control group with caregivers of children that did not have ASD. High internal consistency was observed in both groups. Children in the clinical control group scored much lower on all subscales and on the ATEC as a whole (i.e. their answers were heavily skewed towards less severe behaviour and more advanced development), presumably because those children had already met many developmental milestones compared to children with neurodevelopmental disorders. The large mean differences were still significant after adjusting for several demographic variables. The high reliability and good group validity suggest the ATEC may be a suitable outcome measure for use in interventions in a wider group of neurodevelopmental disorders rather than autism alone. The clinical control group was employed for validation purposes only; we do not suggest that the tool should be used among children with physical health conditions in isolation.

The ATEC is normally used to measure the impact of a given intervention by comparing the scores before and after the intervention. In this study, no intervention was done, and we were instead interested if the tool would show temporal stability with a time window of about 21 days between measurements. We were able to demonstrate very high test–retest reliability, which is consistent with the Brazilian and Iranian studies (Freire et al., 2018; Memari et al., 2013).

Previous studies have demonstrated that the ATEC correlates well with diagnostic tools for ASD (Geier et al., 2013; Magiati et al., 2011). For this reason, we expected that the total ATEC score would correlate with severity of ASD and ID, but only a weak and non-significant correlation was detected. This may be because none of the clinicians who assigned the levels had a specialty training in child psychiatry because such training is not available in Ethiopia, and may thus have had some difficulty interpreting and adhering to the DSM-5 severity rating criteria. Consequently, it can be argued that the severity levels were employed differently than they would have been in a high-income setting. In addition, the severity rating scale used included a 3-point scale and may not have been sufficiently nuanced to allow for any correlations to be identified. Finally, clinician-assigned severity ratings were only available for a subset of children with neurodevelopmental conditions; this modest sample size was insufficient to detect correlations of modest to small effect. We would therefore not interpret our finding as evidence that the adapted ATEC lacks convergent validity. Furthermore, the ATEC correlated moderately and significantly with the PedsQL™ FIM, as hypothesised.

For the PedsQL™ FIM, the results of the CFA indicated that the eight-factor model fitted the data to an acceptable degree. This is consistent with Medrano et al.’s (2013) study where the model fit for the original English version was also acceptable. Our findings, however, are not entirely consistent with the Chinese study (Chen et al., 2011) where the model fit was only marginally acceptable, with the RMSEA not reaching the expected threshold. Internal consistency of the PedsQL™ FIM was mostly good to excellent, which is in line with previous studies (Medrano et al., 2013; Panepinto et al., 2009; Varni et al., 2004). In all instances, the Communication subscale performed the worst, much like in this study. However, we discovered that Cronbach’s alpha in the case group would improve from 0.59 to 0.72 by dropping the third item (‘Was it hard for you to tell doctors and nurses how you feel?’). Further inspection of the distribution of this item showed that almost all participants in both groups answered with *Never* or *Almost never*. We suspect that social desirability might be at play here. Considering that all data collectors were nurses themselves, caregivers may have felt hesitant to endorse this item. We would advise against dropping that item, as its problems are likely an artefact of our data collection method and may be avoided if data collectors are not health professionals.

This study was the first to validate the PedsQL™ FIM in caregivers of children with neurodevelopmental disorders. Since no previous study compared this scale in caregivers with neurodevelopmental disorders versus physical health conditions, we did not have a strong hypothesis regarding expected group differences. The results showed that caregivers of children with neurodevelopmental disorders reported significantly stronger impact of their child’s condition (except for the Cognitive Functioning subscale, where the difference was not significant). We suspect this might be because some of the conditions in the control group were acute and possibly short-term, whereas all neurodevelopmental disorders are typically lifelong. Services for children with neurodevelopmental disorders are limited in Ethiopia; most families receive no formal support and most children, especially those living outside Addis Ababa, are excluded from schools (Tekola et al., 2016; Tilahun et al., 2016). Moreover, families report high levels of stigma and social exclusion (Tekola, Kinfe, et al., 2020; Tilahun et al., 2016). These difficulties impact the quality of life of families and may have contributed further to the markedly lower PedsQL™ FIM scores in the case compared to the clinical control group.

Regarding temporal stability, we reported coefficients mostly in the moderate and occasionally in the good range for all scores and individual items of the PedsQL™ FIM. The ICCs of the scores also had wide intervals, with lower bounds in the poor range. This is not entirely consistent with the Brazilian study (Scarpelli et al., 2008) where higher values were observed and all confidence intervals were narrower. We believe that this discrepancy in findings may again be because of the temporary nature of the health conditions in the control group. The PedsQL™ FIM asks caregivers about problems in the past 7 days; with an average time of 21 days between the first and the second time points, it would be entirely possible that some children in the control group already had their problems resolved prior to the second measurement.

A previous study has shown that the PedsQL™ FIM might be able to discriminate between severities of a given condition (Limbers et al., 2011). Our findings did not support this: severity of ASD and ID correlated weakly and non-significantly with the total score. Still, the correlation was in the expected direction (negative), meaning that higher severity tended to be associated with lower functioning of the caregiver. As reported above, the PedsQL™ FIM also correlated moderately and significantly with the ATEC.

### Limitations

The findings of this study are to be interpreted with certain limitations in mind. First, we used a sample at two hospitals in Addis Ababa, meaning that only help-seeking families who can access the capital city were included. This was also reflected in the sample characteristics (e.g. families were mostly urban residents). Second, our evidence of convergent validity was limited to the association between the two questionnaires. There are no validated autism or ID severity measures available in Amharic, and ratings were conducted by general psychiatrists without specialty training in child psychiatry. The child mental health clinics tend to see primarily more severely affected children, limiting the opportunity for clinicians to gain expertise in diagnosing milder versions of neurodevelopmental disorders. These limitations may have meant our implemented measure of clinician-assigned severity may have had limited value. The final sample consisted of 32 retest participants, which was only about 10% of all participants. For this reason, we were not able to explore differences between the control and case groups during the retest. Finally, this study did not include an evaluation of the sensitivity to change of the instruments in response to intervention.

We were successful in adapting two instruments developed in high-income Western contexts and making them suitable for use in a low-income African context characterised by low literacy and limited awareness about autism and other neurodevelopmental conditions. The ATEC in particular required substantial adaptation. The trickiest section to administer was ATEC subscale 2, which includes some negatively worded items. Future researchers using this instrument in a similar context may wish to consider a further adaptation and rephrase all items to positively worded questions to avoid confusion.

## Conclusion

The ATEC and the PedsQL™ FIM demonstrated adequate reliability and validity after being translated and culturally adapted for the Ethiopian context. The most substantial change introduced in the adaptation process was the use of face-to-face interviews in place of self-administration. The results of our validation work indicate that as long as the adaptation process is extensive and aims to retain conceptual equivalence, both tools can successfully be adapted for use in LMIC settings while still being accessible to people of all literacy levels.

We believe those findings are an important step towards advancing research on neurodevelopmental disorders in Ethiopia, where there has been a lack of validated tools for assessing and monitoring those conditions. Even more established tools should be validated and made accessible to everyone in LMICs, so that the impact of neurodevelopmental disorders on both children and caregivers could be better understood and managed.

## Supplemental Material

sj-docx-1-aut-10.1177_13623613211050751 – Supplemental material for Adaptation and validation of two autism-related measures of skills and quality of life in Ethiopia Supplemental material, sj-docx-1-aut-10.1177_13623613211050751 for Adaptation and validation of two autism-related measures of skills and quality of life in Ethiopia by Anton Borissov, Ioannis Bakolis, Bethlehem Tekola, Mersha Kinfe, Caterina Ceccarelli, Fikirte Girma, Rehana Abdurahman, Tigist Zerihun, Charlotte Hanlon and Rosa A Hoekstra in Autism
