# Supplementary material for: Adaptation and validation of two autism-related measures of skills and quality of life in Ethiopia
Source: Autism. 2021 Dec 7;26(6):1409–22. doi: 10.1177/13623613211050751 (PMC9340138; doi:10.1177/13623613211050751)
Supplement: sj-docx-1-aut-10.1177_13623613211050751 – Supplemental material for Adaptation and validation of two autism-related measures of skills and quality of life in Ethiopia [file sj-docx-1-aut-10.1177_13623613211050751.docx]

# Supplementary Material

This file contains the Amharic versions of the Autism Treatment Evaluation Checklist (ATEC) and the Pediatric Quality of Life Inventory™ Family Impact Module (PedsQL™ FIM), as administered in the study, along with their English equivalents.

For the Sociability subscale of the ATEC, some answer categories have been extended to further clarify the items. Those new suggestions are marked in red and are not part of how the ATEC was administered in our study. However, we recommend that they are included in future administrations. Researchers may also want to consider rephrasing the negatively worded questions of the Sociability subscale (e.g. so that “Is he/she not sociable/approachable?” becomes “Is he/she sociable/approachable?”) to avoid confusion.

This file also contains a number of tables at the bottom, to which we have referred in the main article.

# Autism Treatment Evaluation Checklist (Amharic)

የተ. መለያ ቁጥር **□□□□-□□**

የልጅ ካርድ ቁጥር ጾታ እድሜ ________ የትውልድ ቀን ________

መጠይቁ የሚጠየቀው ሰው ከልጁ ጋር ያለው ግንኙነት ___________________ የዛሬ ቀን __________________

ለሚከተሉት ጥያቄዎች ከተሰጡት አማራጮች ውስጥ መልስ የሚሆነውን አማራጭ ይመልሱ፡፡

**ሀ.ንግግር/ ቋንቋ/ መግባባት**

| 1 | ልጅዎ የራሱን/የራስዋን ስም ያውቃል/ታውቃለች? | አዎ | 2 | SPELANCOMM 1 |
| --- | --- | --- | --- | --- |
|  |  | አይ | 0 |  |
|  |  | አንዳንዴ | 1 |  |
| 2 | ልጅዎ ለአይሆንም ወይም አቁም ምላሽ ይሰጣል/ትሰጣለች? | አዎ | 2 | SPELANCOMM 2 |
|  |  | አይ | 0 |  |
|  |  | አንዳንዴ | 1 |  |
| 3. | ልጅዎ አንዳንድ ትእዛዞችን መከተል ይችላል/ትችላለች? | አዎ | 2 | SPELANCOMM 3 |
|  |  | አይ | 0 |  |
|  |  | አንዳንዴ | 1 |  |
| 4 | ልጅዎ በአንድ ጊዜ ላይ አንድ ቃል መጠቀም ይችላል/ትችላለች?  (አይ!፣ ብሉ፣ ውሀ፣ ወዘተ…)? | አዎ | 2 | SPELANCOMM 4 |
|  |  | አይ | 0 |  |
|  |  | አንዳንዴ | 1 |  |
| 5 | ልጅዎ በአንድ ጊዜ ሁለት ቃላትን መጠቀም ይችላል/ትችላለች?  (እኔ አልፈልግም፣ ቤት ሂዱ)? | አዎ | 2 | SPELANCOMM 5 |
|  |  | አይ | 0 |  |
|  |  | አንዳንዴ | 1 |  |
| 6 | ልጅዎ በአንድ ጊዜ ሶስት ቃላትን መጠቀም ይችላል/ትችላለች | አዎ | 2 | SPELANCOMM 6 |
|  |  | አይ | 0 |  |
|  |  | አንዳንዴ | 1 |  |
| 7 | ልጅዎ አስር ወይም ከዛ በላይ ቃላት ታውቃለች/ያውቃል? | አዎ | 2 | SPELANCOMM 7 |
|  |  | አይ | 0 |  |
|  |  | አንዳንዴ | 1 |  |
| 8 | ልጅዎ አራት ወይም ከዛ በላይ ቃላት ያለው አርፍተ ነገሮች መጠቀም ይችላል/ትችላለች? | አዎ | 2 | SPELANCOMM8 |
|  |  | አይ | 0 |  |
|  |  | አንዳንዴ | 1 |  |
| 9 | ልጅዎ የፈለገውን/የፈለገችውን ነገር ማብራራት ይችላል/ትችላለች? | አዎ | 2 | SPELANCOMM9 |
|  |  | አይ | 0 |  |
|  |  | አንዳንዴ | 1 |  |
| 10 | ልጅዎ ትርጉም ያላቸውን ጥያቄዎች መጠየቅ ይችላል/ትችላለች? | አዎ | 2 | SPELANCOMM10 |
|  |  | አይ | 0 |  |
|  |  | አንዳንዴ | 1 |  |
| 11 | ልጅዎ አግባብነት ያለው ንግግር ማረግ ይችላል/ትችላለች? | አዎ | 2 | SPELANCOMM11 |
|  |  | አይ | 0 |  |
|  |  | አንዳንዴ | 1 |  |
| 12 | ልጅዎ ብዛት ያላቸውን ተከታታይ አረፍተ ነገሮችን መጠቀም ይችላል/ትችላለች? | አዎ | 2 | SPELANCOMM12 |
|  |  | አይ | 0 |  |
|  |  | አንዳንዴ | 1 |  |
| 13 | ልጅዎ ከሰዎች ጋር በንግግር ምልልስ ማካሄድ ይችላል/ትችላለች ? | አዎ | 2 | SPELANCOMM13 |
|  |  | አይ | 0 |  |
|  |  | አንዳንዴ | 1 |  |
| 14 | ልጅዎ በዛ እድሜ እንዳለ ሰው መደበኛ መግባቢያ ችሎታ አለው/አላት? | አዎ | 2 | SPELANCOMM14 |
|  |  | አይ | 0 |  |
|  |  | አንዳንዴ | 1 |  |

ለሚከተሉት ጥያቄዎች ከተሰጡት አማራጮች ውስጥ መልስ የሚሆነውን አማራጭ ይመልሱ፡፡

**ለ. ተግባቢነት**

| 1 | ልጅዎ ከሰው የማይግባባ/የማትግባባ ፣ ለመቅረብ የየማይቻል/የማተቻል ነው/ነች? | አዎ ይግባባል/ትግባባለች ሰውንም ይቀርባል/ትቀርባለች | 0 | SOCIAB1 |
| --- | --- | --- | --- | --- |
|  |  | አይ አይግባባም/አትግባባም  ሰውንም አይቀርብ/አትቀርብም | 2 |  |
|  |  | አንዳንዴ | 1 |  |
| 2 | ልጅዎ ሌሎች ሰዎችን ችላ ይላል/ትላለች? | አዎ ይላል/ትላለች | 2 | SOCIAB2 |
|  |  | አይ አይልም/አትልም | 0 |  |
|  |  | አንዳንዴ | 1 |  |
| 3. | ልጅዎ ሲጠሩት/ሲጠሩአት/ሲያናግሩት/ሲያናግሩአትሰ ምንም ትኩረት አይሰጥም/አትሰጥም ወይም ትንሽ ትኩረት ብቻ ይሰጣል/ትሰጣለች? | አዎ ትንሽ ትኩረት ይሰጣል /ትሰጣለች | 0 | SOCIAB3 |
|  |  | አይ ምንም ትኩረት አይሰጥም/አትሰጥም | 2 |  |
|  |  | አንዳንዴ | 1 |  |
| 4 | ልጅዎ ግትር/እምቢተኛ ነው/ነች ? | አዎ ግትር/እምቢተኛ ነው/ነች? | 2 | SOCIAB4 |
|  |  | አይ ግትር/እምቢተኛ አይደለም? | 0 |  |
|  |  | አንዳንዴ | 1 |  |
| 5 | ልጅዎ የአይን ግንኙነት አያደርግም/አታደርግም? | አዎ ያደርጋል /ታደርጋለች | 0 | SOCIAB5 |
|  |  | አይ አያደርግም/አታደርግም | 2 |  |
|  |  | አንዳንዴ | 1 |  |
| 6 | ልጅዎ ብቻ መተው ይፈልጋል/ትፈልጋለች ? | አዎ ይፈልጋል/ትፈልጋለች | 2 | SOCIAB6 |
|  |  | አይ አይፈልግም/አትፈልግም | 0 |  |
|  |  | አንዳንዴ | 1 |  |
| 7 | ልጅዎ ምንም አይነት ፍቅር አያሳይም/አታሳይም? | አዎ ያሳያል/ታሳያለች | 0 | SOCIAB7 |
|  |  | አይ አያሳይም/አታሳይም | 2 |  |
|  |  | አንዳንዴ | 1 |  |
| 8 | ልጅዎ ቤተሰብ ሰላም አይልም/አትልም? | አዎ ቤተሰብ ሰላም ይላል/ትላለች | 0 | SOCIAB8 |
|  |  | አይ ቤተሰብ ሰላም አይልም/አትልም | 2 |  |
|  |  | አንዳንዴ | 1 |  |
| 9 | ልጅዎ ከሌላ ሰው ጋር ግንኙነት አይፈልግም/አትፈልግም? | አዎ ግንኙነት ይፈልጋል/ትፈልጋለች | 0 | SOCIAB9 |
|  |  | አይ ግንኙነትአይፈልግም/አትፈልግም | 2 |  |
|  |  | አንዳንዴ | 1 |  |
| 10 | ልጅዎ አያስመስልም/አታስመስልም? | አዎ ያስመስላል/ታስመስላለች | 0 | SOCIAB10 |
|  |  | አይ አያስመስልም/አታስመስልም | 2 |  |
|  |  | አንዳንዴ | 1 |  |
| 11 | ልጅዎ መያዝ/መታቀፍ አይፈልግም/አትፈልግም? | አዎ ይፈልጋል /ትፈልጋለች | 0 | SOCIAB11 |
|  |  | አይ አይፈልግም/አትፈልግም | 2 |  |
|  |  | አንዳንዴ | 1 |  |
| 12 | ልጅዎ አያሳይም/አታሳይም ወይም አያካፍልም/አታካፍልም? | አዎ ያሳያል/ታሳያለች | 0 | SOCIAB12 |
|  |  | አይ አያሳይም/አታሳይም | 2 |  |
|  |  | አንዳንዴ | 1 |  |
| 13 | ልጅዎ እጅ አያውለበልብም/አታውለበልብም እንደ .ቻው ቻው? | አዎ ያውለበልባል/ታውለበልባለች | 0 | SOCIAB13 |
|  |  | አይ አያውለበልብም/አታውለበልብም | 2 |  |
|  |  | አንዳንዴ | 1 |  |
| 14 | ልጅዎ አይታዘዝም/ አትታዘዝም እምቢተኛ ነው/ነች? | አዎ ይታዘዛል/ትታዘዛለች | 0 | SOCIAB14 |
|  |  | አይ አይታዘዝም/አትታዘዝም | 2 |  |
|  |  | አንዳንዴ | 1 |  |
| 15 | ልጅዎ ግዜያዊ የሆነ ከፍተኛ ብስጭት/ንዴት/ሀይለኝነት አለው/አላት? | አዎ አለው/አላት | 2 | SOCIAB15 |
|  |  | አይ የለውም/የላትም | 0 |  |
|  |  | አንዳንዴ | 1 |  |
| 16 | ልጅዎ ጓደኛ/ አብሮ የሚሆን የቅርብ ሰው አለው/አላት? | አዎ አለው/አላት | 0 | SOCIAB16 |
|  |  | አይ የለውም/የላትም | 2 |  |
|  |  | አንዳንዴ | 1 |  |
| 17 | ልጅዎ ብዙ ፈገግ አይልም/አትልም? | አዎ ይላል/ትላለች | 0 | SOCIAB17 |
|  |  | አይ አይልም/አትልም | 2 |  |
|  |  | አንዳንዴ | 1 |  |
| 18 | ልጅዎ ለሌሎች ሰዎች ስሜት ይጨነቃል/ትጨነቃለች? | አዎ ይጨነቃል/ትጨነቃለች | 0 | SOCIAB18 |
|  |  | አይ አይጨነቅም/አትጨነቅም | 2 |  |
|  |  | አንዳንዴ | 1 |  |
| 19 | ልጅዎ ሰለመወደድ ግዴየለሽ ነው/ነች? | አዎ ግድየለሽ ነው/ነች | 2 | SOCIAB19 |
|  |  | አይ ግድየለሽ አይደለም/አይደለችም | 0 |  |
|  |  | አንዳንዴ | 1 |  |
| 20 | ልጅዎ ወላጅ/አሳዳጊ ሲሄድ ግዴየለሽ ነው/ነች? | አዎ ግድየለሽ ነው/ነች | 2 | SOCIAB20 |
|  |  | አይ ግድየለሽ አይደለም/አይደለችም | 0 |  |
|  |  | አንዳንዴ | 1 |  |

**ሐ. የስሜት/የመገንዘብ ንቃት፡**

ለሚከተሉት ጥያቄዎች ከተሰጡት አማራጮች ውስጥ መልስ የሚሆነውን አማራጭ ይመልሱ፡፡

ይህንን ምልክት ይጠቀሙ

| 1 | ልጅዎ ስሙ/ስምዋ ሲጠራ ምላሽ ይሰጣል/ትሰጣለች | አዎ | 2 | COGAWAR1 |
| --- | --- | --- | --- | --- |
|  |  | አይ | 0 |  |
|  |  | አንዳንዴ | 1 |  |
| 2 | ልጅዎ ለሙገሳ ምላሽ ይሰጣል/ትሰጣለች | አዎ | 2 | COGAWAR2 |
|  |  | አይ | 0 |  |
|  |  | አንዳንዴ | 1 |  |
| 3. | ልጅዎ ሰዎችን ወይም እንስሳትን ያያል/ታያለች | አዎ | 2 | COGAWAR3 |
|  |  | አይ | 0 |  |
|  |  | አንዳንዴ | 1 |  |
| 4 | ልጅዎ ምስሎችን (እናም ቴሌቨዥን) ያያል/ታያለች | አዎ | 2 | COGAWAR4 |
|  |  | አይ | 0 |  |
|  |  | አንዳንዴ | 1 |  |
| 5 | ልጅዎ ስእሎችን ይስላል/ትስላለች ይቀባል/ትቀባለች | አዎ | 2 | COGAWAR5 |
|  |  | አይ | 0 |  |
|  |  | አንዳንዴ | 1 |  |
| 6 | ልጅዎ በመጫዎቻዎች በተገቢው መንገድ ይጫወታል/ትጫወታለች | አዎ | 2 | COGAWAR6 |
|  |  | አይ | 0 |  |
|  |  | አንዳንዴ | 1 |  |
| 7 | ልጅዎ ተገቢ የሆነ የፊት ገፅታ ማየት ይችላል/ትችላለች | አዎ | 2 | COGAWAR7 |
|  |  | አይ | 0 |  |
|  |  | አንዳንዴ | 1 |  |
| 8 | ልጅዎ ቴሌቨዝን ላይ ያሉ ታሪኮችን ይረዳል/ትረዳለች | አዎ | 2 | COGAWAR8 |
|  |  | አይ | 0 |  |
|  |  | አንዳንዴ | 1 |  |
| 9 | ልጅዎ ማብራሪያዎችን ይረዳል/ትረዳለች | አዎ | 2 | COGAWAR9 |
|  |  | አይ | 0 |  |
|  |  | አንዳንዴ | 1 |  |
| 10 | ልጅዎ ለአካባቢ ግንዛቤ አለው/አላት | አዎ | 2 | COGAWAR10 |
|  |  | አይ | 0 |  |
|  |  | አንዳንዴ | 1 |  |
| 11 | ልጅዎ ለአደጋ ግንዛቤ አለው/አላት | አዎ | 2 | COGAWAR11 |
|  |  | አይ | 0 |  |
|  |  | አንዳንዴ | 1 |  |
| 12 | ልጅዎ በአይነ ህሊና/ምናብ ይስላል/ትስላለች | አዎ | 2 | COGAWAR12 |
|  |  | አይ | 0 |  |
|  |  | አንዳንዴ | 1 |  |
| 13 | ልጅዎ ድርጊቶችን/ክንዋኔዎችን ይጀምራል/ትጀምራለች | አዎ | 2 | COGAWAR13 |
|  |  | አይ | 0 |  |
|  |  | አንዳንዴ | 1 |  |
| 14 | ልጅዎ እራስን ያለብሳል/ታለብሳለች | አዎ | 2 | COGAWAR14 |
|  |  | አይ | 0 |  |
|  |  | አንዳንዴ | 1 |  |
| 15 | ልጅዎ የማወቅ ጉጉት፣ ፍላጎት አለው አላት | አዎ | 2 | COGAWAR15 |
|  |  | አይ | 0 |  |
|  |  | አንዳንዴ | 1 |  |
| 16 | ልጅዎ የማይፈራ /የሚሞክር /ለማወቅ የሚፈልግ ነው | አዎ | 2 | COGAWAR16 |
|  |  | አይ | 0 |  |
|  |  | አንዳንዴ | 1 |  |
| 17 | ልጅዎ በሀሳብ ያተኩራል/ታተኩራለች፤ በሀሳብ ይነጉዳል/ትነጉዳለች | አዎ | 2 | COGAWAR17 |
|  |  | አይ | 0 |  |
|  |  | አንዳንዴ | 1 |  |
| 18 | ልጅዎ ሌሎች ሰዎች ወደሚያዩበት ያያል/ታያለች | አዎ | 2 | COGAWAR18 |
|  |  | አይ | 0 |  |
|  |  | አንዳንዴ | 1 |  |

**መ. ጤና/ ሠውነት/ ባህሪ**

ይህንን ምልክት ይጠቀሙ (ች) ችግር አይደለም

(ት) ትንሽ ችግር

(መ) መካከለኛ ችግር

(ከ) ከባድ ችግር

| 1 | አልጋ ላይ መሽናት | ችግር አይደለም | 0 | BEHAV1 |
| --- | --- | --- | --- | --- |
|  |  | ትንሽ ችግር | 1 |  |
|  |  | መካከለኛ ችግር | 2 |  |
|  |  | ከባድ ችግር | 3 |  |
| 2 | የሽንት ጨርቅ/ዳይፐር | ችግር አይደለም | 0 | BEHAV2 |
|  |  | ትንሽ ችግር | 1 |  |
|  |  | መካከለኛ ችግር | 2 |  |
|  |  | ከባድ ችግር | 3 |  |
| 3. | ሱሪ/የሽንት ጨርቅ/ዳይፐር ላይ ሰገራ ማለት | ችግር አይደለም | 0 | BEHAV3 |
|  |  | ትንሽ ችግር | 1 |  |
|  |  | መካከለኛ ችግር | 2 |  |
|  |  | ከባድ ችግር | 3 |  |
| 4 | ተቅማጥ | ችግር አይደለም | 0 | BEHAV4 |
|  |  | ትንሽ ችግር | 1 |  |
|  |  | መካከለኛ ችግር | 2 |  |
|  |  | ከባድ ችግር | 3 |  |
| 5 | ድርቀት | ችግር አይደለም | 0 | BEHAV5 |
|  |  | ትንሽ ችግር | 1 |  |
|  |  | መካከለኛ ችግር | 2 |  |
|  |  | ከባድ ችግር | 3 |  |
| 6 | የእንቅልፍ ችግር | ችግር አይደለም | 0 | BEHAV6 |
|  |  | ትንሽ ችግር | 1 |  |
|  |  | መካከለኛ ችግር | 2 |  |
|  |  | ከባድ ችግር | 3 |  |
| 7 | ብዙ/ትንሽ መብላት | ችግር አይደለም | 0 | BEHAV7 |
|  |  | ትንሽ ችግር | 1 |  |
|  |  | መካከለኛ ችግር | 2 |  |
|  |  | ከባድ ችግር | 3 |  |
| 8 | እጅግ በጣም የተወሰነ የምግብ አይነት መመገብ መፈለግ | ችግር አይደለም | 0 | BEHAV8 |
|  |  | ትንሽ ችግር | 1 |  |
|  |  | መካከለኛ ችግር | 2 |  |
|  |  | ከባድ ችግር | 3 |  |
| 9 | ቅብጥብጥ | ችግር አይደለም | 0 | BEHAV9 |
|  |  | ትንሽ ችግር | 1 |  |
|  |  | መካከለኛ ችግር | 2 |  |
|  |  | ከባድ ችግር | 3 |  |
| 10 | መልፈስፈስ | ችግር አይደለም | 0 | BEHAV10 |
|  |  | ትንሽ ችግር | 1 |  |
|  |  | መካከለኛ ችግር | 2 |  |
|  |  | ከባድ ችግር | 3 |  |
| 11 | እራስን መምታት/መጉዳት | ችግር አይደለም | 0 | BEHAV11 |
|  |  | ትንሽ ችግር | 1 |  |
|  |  | መካከለኛ ችግር | 2 |  |
|  |  | ከባድ ችግር | 3 |  |
| 12 | ሌሎችን መምታት/መጉዳት | ችግር አይደለም | 0 | BEHAV12 |
|  |  | ትንሽ ችግር | 1 |  |
|  |  | መካከለኛ ችግር | 2 |  |
|  |  | ከባድ ችግር | 3 |  |
| 13 | አጥፊ | ችግር አይደለም | 0 | BEHAV13 |
|  |  | ትንሽ ችግር | 1 |  |
|  |  | መካከለኛ ችግር | 2 |  |
|  |  | ከባድ ችግር | 3 |  |
| 14 | ለድምፆች የተለየ ስሜት ያለው | ችግር አይደለም | 0 | BEHAV14 |
|  |  | ትንሽ ችግር | 1 |  |
|  |  | መካከለኛ ችግር | 2 |  |
|  |  | ከባድ ችግር | 3 |  |
| 15 | የሚጨነቅ/ የሚፈራ | ችግር አይደለም | 0 | BEHAV15 |
|  |  | ትንሽ ችግር | 1 |  |
|  |  | መካከለኛ ችግር | 2 |  |
|  |  | ከባድ ችግር | 3 |  |
| 16 | ደስተኛ ያልሆነ/ የሚያለቅስ | ችግር አይደለም | 0 | BEHAV16 |
|  |  | ትንሽ ችግር | 1 |  |
|  |  | መካከለኛ ችግር | 2 |  |
|  |  | ከባድ ችግር | 3 |  |
| 17 | የሚጥል በሽታ/አዙሪት | ችግር አይደለም | 0 | BEHAV17 |
|  |  | ትንሽ ችግር | 1 |  |
|  |  | መካከለኛ ችግር | 2 |  |
|  |  | ከባድ ችግር | 3 |  |
| 18 | ያለአግባብ የሚደጋገም ንግግር | ችግር አይደለም | 0 | BEHAV18 |
|  |  | ትንሽ ችግር | 1 |  |
|  |  | መካከለኛ ችግር | 2 |  |
|  |  | ከባድ ችግር | 3 |  |
| 19 | የማይለዋወጥ የእለት ተእለት ድርጊቶች/ተግባራት አሉት/አሉዋት | ችግር አይደለም | 0 | BEHAV19 |
|  |  | ትንሽ ችግር | 1 |  |
|  |  | መካከለኛ ችግር | 2 |  |
|  |  | ከባድ ችግር | 3 |  |
| 20 | መጮህ | ችግር አይደለም | 0 | BEHAV20 |
|  |  | ትንሽ ችግር | 1 |  |
|  |  | መካከለኛ ችግር | 2 |  |
|  |  | ከባድ ችግር | 3 |  |
| 21 | የነገሮችን ተመሳሳይነት መፈለግ | ችግር አይደለም | 0 | BEHAV21 |
|  |  | ትንሽ ችግር | 1 |  |
|  |  | መካከለኛ ችግር | 2 |  |
|  |  | ከባድ ችግር | 3 |  |
| 22 | ብዙውን ግዜ የተረበሸ/የተናደደ/የተቅበጠበጠ ባህሪ ማሳየት | ችግር አይደለም | 0 | BEHAV22 |
|  |  | ትንሽ ችግር | 1 |  |
|  |  | መካከለኛ ችግር | 2 |  |
|  |  | ከባድ ችግር | 3 |  |
| 23 | የህመም ስሜት የማይሰማው/የማይሰማት | ችግር አይደለም | 0 | BEHAV23 |
|  |  | ትንሽ ችግር | 1 |  |
|  |  | መካከለኛ ችግር | 2 |  |
|  |  | ከባድ ችግር | 3 |  |
| 24 | የተወሰነ ነገሮች/እቃዎች ይዞ የማይለቅ/የማተለቅ | ችግር አይደለም | 0 | BEHAV24 |
|  |  | ትንሽ ችግር | 1 |  |
|  |  | መካከለኛ ችግር | 2 |  |
|  |  | ከባድ ችግር | 3 |  |
| 25 | ድግግሞሽ ያለው እንቅስቃሴ (መወዛወዝ፣ እጅን ወይም እግር በተመሳሳይ መልኩ ማወዛወዝ) | ችግር አይደለም | 0 | BEHAV25 |
|  |  | ትንሽ ችግር | 1 |  |
|  |  | መካከለኛ ችግር | 2 |  |
|  |  | ከባድ ችግር | 3 |  |

መጠይቁን የሞላው ሰው ስም----------------------- ያረጋገጠው ስም-----------------

ፊርማ----------------------------- ፊርማ-------------------------

ቀን--------------------------------- ቀን-------------------------

*The Amharic adaptation and translation of the ATEC was led by the authors of this manuscript (Borissov et al.). The developers and copyright holders of the original ATEC take no responsibility for the quality of the adaptation and translation.*

# Autism Treatment Evaluation Checklist (English)

ID Code **□□□****□-□□**

Card number of child Gender________

the relationship of the respondent with the child: __________________ Age……………. Date of Birth………….. Today’s Date………….

Please circle the letters to indicate how true each phrase is:

1. **Speech/ Language/ Communication**

| 1 | Does your child know his/her own name? | Yes | 2 | SPELANCOMM 1 |
| --- | --- | --- | --- | --- |
|  |  | No | 0 |  |
|  |  | Sometimes | 1 |  |
| 2 | Does your child respond to ‘no’ or ‘stop’? | Yes | 2 | SPELANCOMM 2 |
|  |  | No | 0 |  |
|  |  | Sometimes | 1 |  |
| 3. | Can your child follow some commands? | Yes | 2 | SPELANCOMM 3 |
|  |  | No | 0 |  |
|  |  | Sometimes | 1 |  |
| 4 | Can your child use one word at a time (NO! Eat, Water, etc)? | Yes | 2 | SPELANCOMM 4 |
|  |  | No | 0 |  |
|  |  | Sometimes | 1 |  |
| 5 | Can your child use 2 words at a time  (Don’t want, Go home, etc)? | Yes | 2 | SPELANCOMM 5 |
|  |  | No | 0 |  |
|  |  | Sometimes | 1 |  |
| 6 | Can your child use 3 words at a time? | Yes | 2 | SPELANCOMM 6 |
|  |  | No | 0 |  |
|  |  | Sometimes | 1 |  |
| 7 | Does your child know 10 or more words? | Yes | 2 | SPELANCOMM 7 |
|  |  | No | 0 |  |
|  |  | Sometimes | 1 |  |
| 8 | Can your child use sentences with 4 or more words? | Yes | 2 | SPELANCOMM8 |
|  |  | No | 0 |  |
|  |  | Sometimes | 1 |  |
| 9 | Can he/she explains what he/she wants? | Yes | 2 | SPELANCOMM9 |
|  |  | No | 0 |  |
|  |  | Sometimes | 1 |  |

| 10 | Does he/she ask meaningful questions? | Yes | 2 | SPELANCOMM10 |
| --- | --- | --- | --- | --- |
|  |  | No | 0 |  |
|  |  | Sometimes | 1 |  |
| 11 | Does her speech tend to be meaningful/ relevant? | Yes | 2 | SPELANCOMM11 |
|  |  | No | 0 |  |
|  |  | Sometimes | 1 |  |
| 12 | Does he/she often use several successive sentences? | Yes | 2 | SPELANCOMM12 |
|  |  | No | 0 |  |
|  |  | Sometimes | 1 |  |
| 13 | Can he/she carry on fairly good conversations? | Yes | 2 | SPELANCOMM13 |
|  |  | No | 0 |  |
|  |  | Sometimes | 1 |  |
| 14 | Does he/she have a normal ability to communicate for his/her age? | Yes | 2 | SPELANCOMM14 |
|  |  | No | 0 |  |
|  |  | Sometimes | 1 |  |

1. **Sociability:**

| 1 | Is he/she not sociable/approachable? | Yes, s/he is sociable/approachable | 0 | SOCIAB1 |
| --- | --- | --- | --- | --- |
|  |  | No, s/he is not sociable/approachable | 2 |  |
|  |  | Sometimes | 1 |  |
| 2 | Does he/she ignore other people? | Yes, s/he does | 2 | SOCIAB2 |
|  |  | No, s/he doesn’t | 0 |  |
|  |  | Sometimes | 1 |  |
| 3 | Does he/she pay no attention when addressed or pays little attention? | Yes, s/he does pay attention | 0 | SOCIAB3 |
|  |  | No, s/he doesn’t pay attention | 2 |  |
|  |  | Sometimes | 1 |  |
| 4 | Is he/she uncooperative and resistant? | Yes, s/he is resistant | 2 | SOCIAB4 |
|  |  | No, s/he isn’t resistant | 0 |  |
|  |  | Sometimes | 1 |  |
| 5 | Does he/she make no eye contact? | Yes, s/he does make eye contact | 0 | SOCIAB5 |
|  |  | No, s/he doesn’t make eye contact | 2 |  |
|  |  | Sometimes | 1 |  |
| 6 | Does he/she prefer to be left alone? | Yes, s/he does prefer | 2 | SOCIAB6 |
|  |  | No, s/he doesn’t prefer | 0 |  |
|  |  | Sometimes | 1 |  |
| 7 | Does he/she show no affection? | Yes, s/he does show affection | 0 | SOCIAB7 |
|  |  | No, s/he doesn’t show affection | 2 |  |
|  |  | Sometimes | 1 |  |
| 8 | He/she does not greet parents? | Yes, s/he does greet parents | 0 | SOCIAB8 |
|  |  | No, s/he doesn’t greet parents | 2 |  |
|  |  | Sometimes | 1 |  |
| 9 | He/she does not want contact with others? | Yes, s/he does want contact | 0 | SOCIAB9 |
|  |  | No, s/he doesn’t want contact | 2 |  |
|  |  | Sometimes | 1 |  |
| 10 | He/she does not imitate? | Yes, s/he does imitate | 0 | SOCIAB10 |
|  |  | No, s/he doesn’t imitate | 2 |  |
|  |  | Sometimes | 1 |  |
| 11 | He/she does not want to be held/cuddled? | Yes, s/he does want | 0 | SOCIAB11 |
|  |  | No, s/he doesn’t want | 2 |  |
|  |  | Sometimes | 1 |  |
| 12 | He/she does not share or show? | Yes, s/he does show | 0 | SOCIAB12 |
|  |  | No, s/he doesn’t show | 2 |  |
|  |  | Sometimes | 1 |  |
| 13 | He/she does not wave 'bye bye'? | Yes, s/he does wave | 0 | SOCIAB13 |
|  |  | No, s/he doesn’t wave | 2 |  |
|  |  | Sometimes | 1 |  |
| 14 | Is he/she disagreeable/not compliant? | Yes, s/he is compliant | 0 | SOCIAB14 |
|  |  | No, s/he is not compliant | 2 |  |
|  |  | Sometimes | 1 |  |
| 15 | Does he/she have temper tantrums? | Yes, s/he does have | 2 | SOCIAB15 |
|  |  | No, s/he doesn’t have | 0 |  |
|  |  | Sometimes | 1 |  |
| 16 | Does he/she have friends/companions? | Yes, s/he does have | 0 | SOCIAB16 |
|  |  | No, s/he doesn’t have | 2 |  |
|  |  | Sometimes | 1 |  |
| 17 | He/she does not smile a lot? | Yes, s/he does smile | 0 | SOCIAB17 |
|  |  | No, s/he doesn’t smile | 2 |  |
|  |  | Sometimes | 1 |  |
| 18 | Is he/she sensitive to other's feelings? | Yes, s/he is sensitive | 0 | SOCIAB18 |
|  |  | No, s/he is not sensitive | 2 |  |
|  |  | Sometimes | 1 |  |
| 19 | Is he/she indifferent to being liked? | Yes, s/he is indifferent | 2 | SOCIAB19 |
|  |  | No, s/he isn’t indifferent | 0 |  |
|  |  | Sometimes | 1 |  |
| 20 | Is he/she indifferent if parent(s) leave? | Yes, s/he is indifferent | 2 | SOCIAB20 |
|  |  | No, s/he isn’t indifferent | 0 |  |
|  |  | Sometimes | 1 |  |

1. **Sensory /Cognitive Awareness:**

| 1 | Re  Does your child respond to his/her own name? | Yes | 2 | COGAWAR1 |
| --- | --- | --- | --- | --- |
|  |  | No | 0 |  |
|  |  | Sometimes | 1 |  |
| 2 | Does your child respond to praise? | Yes | 2 | COGAWAR2 |
|  |  | No | 0 |  |
|  |  | Sometimes | 1 |  |
| 3. | Does your child look at people and animals? | Yes | 2 | COGAWAR3 |
|  |  | No | 0 |  |
|  |  | Sometimes | 1 |  |
| 4 | Does your child look at pictures (and T.V.)? | Yes | 2 | COGAWAR4 |
|  |  | No | 0 |  |
|  |  | Sometimes | 1 |  |
| 5 | Does your child do drawing, coloring, art? | Yes | 2 | COGAWAR5 |
|  |  | No | 0 |  |
|  |  | Sometimes | 1 |  |
| 6 | Does your child play with toys appropriately? | Yes | 2 | COGAWAR6 |
|  |  | No | 0 |  |
|  |  | Sometimes | 1 |  |
| 7 | Does your child have appropriate facial expression? | Yes | 2 | COGAWAR7 |
|  |  | No | 0 |  |
|  |  | Sometimes | 1 |  |
| 8 | Does your child understand stories on T.V.? | Yes | 2 | COGAWAR8 |
|  |  | No | 0 |  |
|  |  | Sometimes | 1 |  |
| 9 | Does your child understand explanations? | Yes | 2 | COGAWAR9 |
|  |  | No | 0 |  |
|  |  | Sometimes | 1 |  |
| 10 | Is your child aware of the environment? | Yes | 2 | COGAWAR10 |
|  |  | No | 0 |  |
|  |  | Sometimes | 1 |  |
| 11 | Is your child aware of danger? | Yes | 2 | COGAWAR11 |
|  |  | No | 0 |  |
|  |  | Sometimes | 1 |  |
| 12 | Does your child show imagination? | Yes | 2 | COGAWAR12 |
|  |  | No | 0 |  |
|  |  | Sometimes | 1 |  |
| 13 | Does your child initiate activities? | Yes | 2 | COGAWAR13 |
|  |  | No | 0 |  |
|  |  | Sometimes | 1 |  |
| 14 | Does your child dress himself/herself? | Yes | 2 | COGAWAR14 |
|  |  | No | 0 |  |
|  |  | Sometimes | 1 |  |
| 15 | Is your child curious, interested? | Yes | 2 | COGAWAR15 |
|  |  | No | 0 |  |
|  |  | Sometimes | 1 |  |
| 16 | Is your child venturesome, does he/she explore? | Yes | 2 | COGAWAR16 |
|  |  | No | 0 |  |
|  |  | Sometimes | 1 |  |
| 17 | Is your child “tuned in”/not spacey? | Yes | 2 | COGAWAR17 |
|  |  | No | 0 |  |
|  |  | Sometimes | 1 |  |
| 18 | Does your child look where others are looking? | Yes | 2 | COGAWAR18 |
|  |  | No | 0 |  |
|  |  | Sometimes | 1 |  |

1. **Health/Physical/Behavior:**

**Use this code:**

**[N] Not a Problem**

**[MI] Minor Problem**

**[MO] Moderate Problem**

**[S] Serious Problem**

| 1 | Be Bed-wetting | N | 0 | BEHAV1 |
| --- | --- | --- | --- | --- |
|  |  | MI | 1 |  |
|  |  | MO | 2 |  |
|  |  | S | 3 |  |
| 2 | Wets pants/diapers | N | 0 | BEHAV2 |
|  |  | MI | 1 |  |
|  |  | MO | 2 |  |
|  |  | S | 3 |  |
| 3. | Soils pants/diapers | N | 0 | BEHAV3 |
|  |  | MI | 1 |  |
|  |  | MO | 2 |  |
|  |  | S | 3 |  |
| 4 | Diarrhea | N | 0 | BEHAV4 |
|  |  | MI | 1 |  |
|  |  | MO | 2 |  |
|  |  | S | 3 |  |
| 5 | Constipation | N | 0 | BEHAV5 |
|  |  | MI | 1 |  |
|  |  | MO | 2 |  |
|  |  | S | 3 |  |
| 6 | Sleep problems | N | 0 | BEHAV6 |
|  |  | MI | 1 |  |
|  |  | MO | 2 |  |
|  |  | S | 3 |  |
| 7 | Eats too much/too little | N | 0 | BEHAV7 |
|  |  | MI | 1 |  |
|  |  | MO | 2 |  |
|  |  | S | 3 |  |
| 8 | Extremely limited diet | N | 0 | BEHAV8 |
|  |  | MI | 1 |  |
|  |  | MO | 2 |  |
|  |  | S | 3 |  |
| 9 | Hyperactive | N | 0 | BEHAV9 |
|  |  | MI | 1 |  |
|  |  | MO | 2 |  |
|  |  | S | 3 |  |
| 10 | Lethargic | N | 0 | BEHAV10 |
|  |  | MI | 1 |  |
|  |  | MO | 2 |  |
|  |  | S | 3 |  |
| 11 | Hits or injures self | N | 0 | BEHAV11 |
|  |  | MI | 1 |  |
|  |  | MO | 2 |  |
|  |  | S | 3 |  |
| 12 | Hits or injures others | N | 0 | BEHAV12 |
|  |  | MI | 1 |  |
|  |  | MO | 2 |  |
|  |  | S | 3 |  |
| 13 | Destructive | N | 0 | BEHAV13 |
|  |  | MI | 1 |  |
|  |  | MO | 2 |  |
|  |  | S | 3 |  |
| 14 | Sound-sensitive | N | 0 | BEHAV14 |
|  |  | MI | 1 |  |
|  |  | MO | 2 |  |
|  |  | S | 3 |  |
| 15 | Anxious/fearful | N | 0 | BEHAV15 |
|  |  | MI | 1 |  |
|  |  | MO | 2 |  |
|  |  | S | 3 |  |
| 16 | Unhappy/crying | N | 0 | BEHAV16 |
|  |  | MI | 1 |  |
|  |  | MO | 2 |  |
|  |  | S | 3 |  |
| 17 | Seizures | N | 0 | BEHAV17 |
|  |  | MI | 1 |  |
|  |  | MO | 2 |  |
|  |  | S | 3 |  |
| 18 | Obsessive speech | N | 0 | BEHAV18 |
|  |  | MI | 1 |  |
|  |  | MO | 2 |  |
|  |  | S | 3 |  |
| 19 | Rigid routines | N | 0 | BEHAV19 |
|  |  | MI | 1 |  |
|  |  | MO | 2 |  |
|  |  | S | 3 |  |
| 20 | Shouts or screams | N | 0 | BEHAV20 |
|  |  | MI | 1 |  |
|  |  | MO | 2 |  |
|  |  | S | 3 |  |
| 21 | Demands sameness | N | 0 | BEHAV21 |
|  |  | MI | 1 |  |
|  |  | MO | 2 |  |
|  |  | S | 3 |  |
| 22 | Often agitated | N | 0 | BEHAV22 |
|  |  | MI | 1 |  |
|  |  | MO | 2 |  |
|  |  | S | 3 |  |
| 23 | Not sensitive to pain | N | 0 | BEHAV23 |
|  |  | MI | 1 |  |
|  |  | MO | 2 |  |
|  |  | S | 3 |  |
| 24 | "Hooked" or Fixated on certain objects/topics | N | 0 | BEHAV24 |
|  |  | MI | 1 |  |
|  |  | MO | 2 |  |
|  |  | S | 3 |  |
| 25 | Repetitive movements (stimming, rocking, etc.) | N | 0 | BEHAV25 |
|  |  | MI | 1 |  |
|  |  | MO | 2 |  |
|  |  | S | 3 |  |

Form completed by…………………………………………….

*The Amharic adaptation and translation of the ATEC was led by the authors of this manuscript (Borissov et al.). The developers and copyright holders of the original ATEC take no responsibility for the quality of the adaptation and translation.*

# PedsQL™ Family Impact Module (Amharic)

የተ. መለያ ቁጥር □□□□-□□

| መመሪያ  አንዳንድ ጊዜ የልጆችን ጤና አስመልክቶ የቤተሰቡን ልዩ ትኩረት የሚፈልጉና የሚያስቸግሩ የጤና ጉዳዮች አሉ፡፡ በሚከተለው ገጽ እርስዎን ሊያስቸግሩ የሚችሉ ዝርዝር ጉዳዮች አሉ፣ እባክዎትን ባለፈው አንድ ሳምንት ውስጥ በእያንዳነዱ ጉዳይ እርስዎ ምን ያህል ይቸገሩ እንደነበር በማክበብ ይግለጹ፡፡  0 በፍጹም ችግር ካልነበረው ዜሮን ያክብቡ  1 ችግሩ ያን ያህል ችግር የሚባል አይደለም የሚሉ ከሆነ አንድን ያክብቡ  2 አንዳንድ ጊዜ ችግር ከነበረው ሁለትን ያክብቡ  3 ብዙ ጊዜ ችግር ከነበረው ሶስትን ያክብቡ  4 ሁል ጊዜ ችግር ከነበረው አራትን ያክብቡ  ትክክል ወይም የተሳሳተ መልስ የለም  ያልተረዱት ጥያቄ ካለ ለተጨማሪ ማብራሪያ እገዛ ይጠይቁ |
| --- |

በልጅዎ ጤና ምክንያት ባለፈው አንድ ሳምንት ውስጥ ቀጥለው በተዘረዘሩት ጉዳዮች ምን ያህል ይቸገሩ ነበር ……

| **አካላዊ ሁኔታ ( ቀጥለው ከተዘረዘሩት ጋር ያለው ችግር …)** | **በፍጹም** | **ችግር የሚባል አይደለም** | **አንዳ ንድ ጊዜ** | **ብዙ ጊዜ** | **ሁል ጊዜ** |  |
| --- | --- | --- | --- | --- | --- | --- |
| 1. ቀን ቀን ድካም ይሰማዎታል? | 0 | 1 | 2 | 3 | 4 | PHYSFUNC 1 |
| 2. ጠዋት ሲነቁ የድካም ስሜት ይሰማዎት ነበር? | 0 | 1 | 2 | 3 | 4 | PHYSFUNC 2 |
| 3. የሚፈልጉአቸውን ነገሮች ለመስራት በጣም የድካም ስሜት ይሰማዎት ነበር? | 0 | 1 | 2 | 3 | 4 | PHYSFUNC 3 |
| 4. ራስዎትን ያሞታል? | 0 | 1 | 2 | 3 | 4 | PHYSFUNC 4 |
| 5. ሰውትዎትን ይደክምዎት ነበር? | 0 | 1 | 2 | 3 | 4 | PHYSFUNC 5 |
| 6. ሆድዎትን ያመዎታል? | 0 | 1 | 2 | 3 | 4 | PHYSFUNC 6 |

| **ስሜታዊ ሁኔታ ( ቀጥለው ከተዘረዘሩት ጋር ያለው ችግር … )** | **በፍጹም** | **ችግር የሚባል አይደለም** | **አንዳ ንድ ጊዜ** | **ብዙ ጊዜ** | **ሁል ጊዜ** |  |
| --- | --- | --- | --- | --- | --- | --- |
| 1. የመጨነቅ ስሜት ይሰማዎት ነበር? | 0 | 1 | 2 | 3 | 4 | EMOFUNC1 |
| 2. የሃዘን ስሜት ይሰማዎት ነበር? | 0 | 1 | 2 | 3 | 4 | EMOFUNC2 |
| 3. የንዴት ስሜት ይሰማዎት ነበር? | 0 | 1 | 2 | 3 | 4 | EMOFUNC3 |
| 4. የመበሳጫት/የመማረር ስሜት ይሰማዎት ነበር? | 0 | 1 | 2 | 3 | 4 | EMOFUNC4 |
| 5. ራሶን ያለመርዳትና ተስፋ የመቁረጥ ስሜት ይሰማዎት ነበር? | 0 | 1 | 2 | 3 | 4 | EMOFUNC5 |

| **ማህበራዊ ጉዳዮች ( ቀጥለው ከተዘረዘሩት ጋር ያለው ችግር … )** | **በፍጹም** | **ችግር የሚባል አይደለም** | **አንዳ ንድ ጊዜ** | **ብዙ ጊዜ** | **ሁል ጊዜ** |  |
| --- | --- | --- | --- | --- | --- | --- |
| 1. ከሌሎች ሰዎች የመነጠል ስሜት ይሰማዎት ነበር? | 0 | 1 | 2 | 3 | 4 | SOCFUNC1 |
| 2. ከሌሎች ሰዎች ድጋፍ ማግኘት አይችሉም ነበር? | 0 | 1 | 2 | 3 | 4 | SOCFUNC2 |
| 3. ለማህበራዊ ተግባራት የሚሆን ጊዜ ያለማግኘት ችግር ነበረቦት? | 0 | 1 | 2 | 3 | 4 | SOCFUNC3 |
| 4. ማህበራዊ ተግባራትን ለመከወን በቂ የሆነ ሃይል የማጣት ችግር ነበረቦት? | 0 | 1 | 2 | 3 | 4 | SOCFUNC4 |

| **ነገሮችን የመገንዘብ ሁኔታ ( ቀጥለው ከተዘረዘሩት ጋር ያለው ችግር … )** | **በፍጹም** | **ችግር የሚባል አይደለም** | **አንዳ ንድ ጊዜ** | **ብዙ ጊዜ** | **ሁል ጊዜ** |  |
| --- | --- | --- | --- | --- | --- | --- |
| 1. በነገሮች ላይ ትኩረት መስጠት ይከብዶት ነበር? | 0 | 1 | 2 | 3 | 4 | COGNFUNC1 |
| 2. ሰዎች የነገሩዎትን ነገር ለማስታወስ ይከብዶት ነበር? | 0 | 1 | 2 | 3 | 4 | COGNFUNC2 |
| 3. የሰሙትን ነገር ለማስታወስ ይከብዶት ነበር? | 0 | 1 | 2 | 3 | 4 | COGNFUNC3 |
| 4. በፍጥነት ማሰብ ይከብዶት ነበር? | 0 | 1 | 2 | 3 | 4 | COGNFUNC4 |
| 5. ቀደም ብለው ሲያስቡ የነበረውን ነገር ለማስታወስ ይከብዶት ነበር? |  |  |  |  |  | COGNFUNC5 |

| **ተግባቦት ( ቀጥለው ከተዘረዘሩት ጋር ያለው ችግር … )** | **በፍጹም** | **ችግር የሚባል አይደለም** | **አንዳ ንድ ጊዜ** | **ብዙ ጊዜ** | **ሁል ጊዜ** |  |
| --- | --- | --- | --- | --- | --- | --- |
| 1.ሌሎች ሰዎች የቤተሰብዎትን ሁኔታ የማይረዱ ይመስሎት ነበር? | 0 | 1 | 2 | 3 | 4 | COMM1 |
| 2. የልጅዎን የጤና ሁኔታ ከሌሎች ሰዎች ጋር ለማውራት ይቸገሩ ነበር? | 0 | 1 | 2 | 3 | 4 | COMM2 |
| 3. ምን እንደሚሰማዎት ለዶክተሮችና ለነርሶች መናገር ይከብዶት ነበር? | 0 | 1 | 2 | 3 | 4 | COMM3 |

በልጅዎ ጤና ም ክንያት ባለፈው አንድ ሳምንት ውስጥ ቀጥለው በ ተዘረዘሩት መስፈርቶች ምን ያህል ይቸገሩ ነበር …

| **ሥጋት ( ቀጥለው ከተዘረዘሩት ጋር ያለው ችግር … ) …)** | **በፍጹም** | **ችግር የሚባል አይደለም** | **አንዳ ንድ ጊዜ** | **ብዙ ጊዜ** | **ሁል ጊዜ** |  |
| --- | --- | --- | --- | --- | --- | --- |
| 1. የልጅዎ ህክምና ውጤታማ መሆን አለመሆኑ ያሰጋዎት ነበር? | 0 | 1 | 2 | 3 | 4 | WOR1 |
| 2. ከልጅዎ ህክምና ጋር ተያይዞ ያለው የጎንዮሽ ጉዳት ያሰጋዎት ነበር? | 0 | 1 | 2 | 3 | 4 | WOR2 |
| 3. ሌሎች ሰዎች ስለልጅዎ ሁኔታ ያላቸው አጸፋዊ ምላሽ ያስጨንቆት ነበር? | 0 | 1 | 2 | 3 | 4 | WOR3 |
| 4. የልጅዎ በሽታ በሌሎች የቤተሰቡ አባላት ላይ ያለው ተጽእኖ ያስጨንቆት ነበር? | 0 | 1 | 2 | 3 | 4 | WOR4 |
| 5. የልጅዎ የወደፊት ህይወት ያሰጋዎታል? | 0 | 1 | 2 | 3 | 4 | WOR5 |

| መመሪያ ከዚህ በታች የርስዎን ቤተሰብ ሊያስቸግሩ የሚችሉ ዝርዝር ጉዳዮች አሉ፣ እባክዎትን ባለፈው አንድ ሳምንት ውስጥ እያንዳንዱ ጉዳይ የርስዎን ቤተሰብ ምን ያህል ያስቸግር እንደነበር ይግለጹልኝ፡፡ |
| --- |

በልጅዎ ጤና ም ክንያት ባለፈው አንድ ሳምንት ውስጥ ቀጥለው ባሉት መስፈርቶች ቤተሰብዎ ምን ያህል ይቸገር እንደነበር …

| **የዕለት ተዕለት እንቅስቃሴ ( ቀጥለው ከተዘረዘሩት ጋር ያለው ችግር … ) )** | **በፍጹም** | **ችግር የሚባል አይደለም** | **አንዳ ንድ ጊዜ** | **ብዙ ጊዜ** | **ሁል ጊዜ** |  |
| --- | --- | --- | --- | --- | --- | --- |
| 1. የቤት ውስጥ ስራዎች ብዙ ጊዜና ጉልበት የመውሰድ ነገር ነበር? | 0 | 1 | 2 | 3 | 4 | DAILACT1 |
| 2. የቤት ውስጥ ስራዎችን ለመጨረስ የሚያስችል ጊዜ አለማግኘት ችግር ነበር? | 0 | 1 | 2 | 3 | 4 | DAILACT2 |
| 3. የቤት ውስጥ ስራዎችን ለመጨረስ ከባድ የድካም ስሜት ነበር? | 0 | 1 | 2 | 3 | 4 | DAILACT3 |

| **ቤተሰባዊ ግንኙነት ( ቀጥለው ከተዘረዘሩት ጋር ያለው ችግር … )** | **በፍጹም** | **ችግር የሚባል አይደለም** | **አንዳ ንድ ጊዜ** | **ብዙ ጊዜ** | **ሁል ጊዜ** |  |
| --- | --- | --- | --- | --- | --- | --- |
| 1. በቤተሰቡ አባላት መካከል የመግባባት ችግር ነበር? | 0 | 1 | 2 | 3 | 4 | FAMREL1 |
| 2. በቤተሰቡ አባላት መካከል ግጭት ተከስቶ ነበር? | 0 | 1 | 2 | 3 | 4 | FAMREL2 |
| 3. እንደቤተሰብ በጋራ ውሳኔ ለመወሰን የመቸገር ነገር ነበር? | 0 | 1 | 2 | 3 | 4 | FAMREL3 |
| 4. የቤተሰቡን ችግር በጋራ ለመፍታት የመቸገር ነገር ነበር? | 0 | 1 | 2 | 3 | 4 | FAMREL4 |
| 5. በቤተሰቡ አባላት መካከል ጭንቀት ወይም ውጥረት ነበር? | 0 | 1 | 2 | 3 | 4 | FAMREL5 |

መጠይቁን የሞላው ሰው ያረጋገጠው ሰው

ስም ስም

ፊርማ ፊርማ

ቀን ቀን

*PedsQL™ contact information and permission to use: Mapi Research Trust, Lyon, France. Internet: https://eprovide.mapi‐trust.org and http://www.pedsql.org*

# PedsQL™ Family Impact Module (English)

| **DIRECTIONS**  Sometimes in relation to children’s health there are health issues which need family’s special attention and which are difficult. On the following page is a list of things that might be a problem for **you**. Please tell us **how** **much of a problem** each one has been for **you** during the **last 7 days** by circling:  **0** if it is **never** a problem **circle 0**  **1** if the problem is **almost never** a problem **circle 1**  **2** if it is **Sometimes** a problem circle 2  **3** if it is **often** a problem circle 3  **4** if it is **almost always** a problem circle 4  There are no right or wrong answers.  If you do not understand a question, please ask for help. |
| --- |

*In the last 7 days, as a result of your child’s health, how much of a problem have* ***you*** *had with…*

| **Physical Functioning *(problems with…)*** | **Never** | **Almost Never** | **Some-times** | **Often** | **Almost**  **Always** |  |
| --- | --- | --- | --- | --- | --- | --- |
| 1. Did you feel tired during the day? | 0 | 1 | 2 | 3 | 4 | PHYSFUNC 1 |
| 1. Did you feel tired when you wake up in the morning? | 0 | 1 | 2 | 3 | 4 | PHYSFUNC 2 |
| 1. Did you feel too tired to do the things you like to do? | 0 | 1 | 2 | 3 | 4 | PHYSFUNC 3 |
| 1. Did you get headaches? | 0 | 1 | 2 | 3 | 4 | PHYSFUNC 4 |
| 1. Did you feel physically weak? | 0 | 1 | 2 | 3 | 4 | PHYSFUNC 5 |
| 1. Did you feel sick to your stomach? | 0 | 1 | 2 | 3 | 4 | PHYSFUNC 6 |

| **Emotional Functioning *(problems with…)*** | **Never** | **Almost Never** | **Some-times** | **Often** | **Almost**  **Always** |  |
| --- | --- | --- | --- | --- | --- | --- |
| 1. Did you feel anxious? | 0 | 1 | 2 | 3 | 4 | EMOFUNC1 |
| 1. Did you feel sad? | 0 | 1 | 2 | 3 | 4 | EMOFUNC2 |
| 1. Did you feel angry? | 0 | 1 | 2 | 3 | 4 | EMOFUNC3 |
| 1. Did you feel frustrated? | 0 | 1 | 2 | 3 | 4 | EMOFUNC4 |
| 1. Did you feel helpless or hopeless? | 0 | 1 | 2 | 3 | 4 | EMOFUNC5 |
| **Social Functioning *(problems with…)*** | **Never** | **Almost Never** | **Some-times** | **Often** | **Almost**  **Always** |  |
| 1. Did you feel isolated from others? | 0 | 1 | 2 | 3 | 4 | SOCFUNC1 |
| 1. Did you have trouble getting support from others? | 0 | 1 | 2 | 3 | 4 | SOCFUNC2 |
| 1. Did you find it hard to find time for social activities? | 0 | 1 | 2 | 3 | 4 | SOCFUNC3 |
| 1. Did you have enough energy for social activities? | 0 | 1 | 2 | 3 | 4 | SOCFUNC4 |

| **Cognitive Functioning *(problems with…)*** | **Never** | **Almost Never** | **Some-times** | **Often** | **Almost**  **Always** |  |
| --- | --- | --- | --- | --- | --- | --- |
| 1. was it hard for you to keep your attention on things? | 0 | 1 | 2 | 3 | 4 | COGNFUNC1 |
| 2. Was it hard for you to remember what people tell you? | 0 | 1 | 2 | 3 | 4 | COGNFUNC2 |
| 3. Was it hard for you to remember what you just heard? | 0 | 1 | 2 | 3 | 4 | COGNFUNC3 |
| 4. Was it hard for you to think quickly? | 0 | 1 | 2 | 3 | 4 | COGNFUNC4 |
| 5. Did you have trouble remembering what you were just thinking? | 0 | 1 | 2 | 3 | 4 | COGNFUNC5 |

| **Communication *(problems with…)*** | **Never** | **Almost Never** | **Some-times** | **Often** | **Almost**  **Always** |  |
| --- | --- | --- | --- | --- | --- | --- |
| 1. Did you feel that others do not understand your family’s situation? | 0 | 1 | 2 | 3 | 4 | COMM1 |
| 2. Was it hard for you to talk about your child’s health with others? | 0 | 1 | 2 | 3 | 4 | COMM2 |
| 3. Was it hard for you to tell doctors and nurses how you feel? | 0 | 1 | 2 | 3 | 4 | COMM3 |

*In the last 7 days, as a result of your child’s health, how much of a problem have* ***you*** *had with…*

| **Worry *(problems with…)*** | **Never** | **Almost Never** | **Some-times** | **Often** | **Almost**  **Always** |  |
| --- | --- | --- | --- | --- | --- | --- |
| 1. Were you worried about whether or not your child’s medical treatments are working? | 0 | 1 | 2 | 3 | 4 | WOR1 |
| 1. Were you worried about the side effects of your child’s medications/medical treatments? | 0 | 1 | 2 | 3 | 4 | WOR2 |
| 1. Were you worried about how others will react to your child’s condition? | 0 | 1 | 2 | 3 | 4 | WOR3 |
| 1. Were you worried about how your child’s illness is affecting other family members? | 0 | 1 | 2 | 3 | 4 | WOR4 |
| 1. Were you worried about your child’s future? | 0 | 1 | 2 | 3 | 4 | WOR5 |

| **DIRECTIONS**  Below is a list of things that might be a problem for **your family**. Please tell us **how** **much of a problem** each one has been for **your family** during the **last 7 days.** |
| --- |

*In the last 7 days, as a result of your child’s health, how much of a problem has* ***your family*** *had with…*

| **Daily Activities *(problems with…)*** | **Never** | **Almost Never** | **Some-times** | **Often** | **Almost**  **Always** |  |
| --- | --- | --- | --- | --- | --- | --- |
| 1. Were family activities taking more time and effort? | 0 | 1 | 2 | 3 | 4 | DAILACT1 |
| 1. Was there difficulty finding time to finish household tasks? | 0 | 1 | 2 | 3 | 4 | DAILACT2 |
| 1. Feeling too tired to finish household tasks Were you feeling too tired to finish household tasks? | 0 | 1 | 2 | 3 | 4 | DAILACT3 |

| **Family Relationships *(problems with…)*** | **Never** | **Almost Never** | **Some-times** | **Often** | **Almost**  **Always** |  |
| --- | --- | --- | --- | --- | --- | --- |
| 1. Was there lack of communication between family members? | 0 | 1 | 2 | 3 | 4 | FAMREL1 |
| 1. Were there conflicts between family members? | 0 | 1 | 2 | 3 | 4 | FAMREL2 |
| 1. Was there difficulty making decisions together as a family? | 0 | 1 | 2 | 3 | 4 | FAMREL3 |
| 1. Was there difficulty solving family problems together? | 0 | 1 | 2 | 3 | 4 | FAMREL4 |
| 1. Was there stress or tension between family members? 2. significant others | 0 | 1 | 2 | 3 | 4 | FAMREL5 |

Form completed by Form checked by

Name Name

Signature Signature

Date Date

*PedsQL™ contact information and permission to use: Mapi Research Trust, Lyon, France. Internet: https://eprovide.mapi‐trust.org and http://www.pedsql.org*

| Table A.1 |  |
| --- | --- |
| *Physical health conditions in the control group (n = 139)* | |
| Condition | Count |
| Severe community-acquired pneumonia | 22 |
| Segmental arterial mediolysis | 7 |
| Congestive heart failure | 6 |
| Asthma | 6 |
| Acute Gastroenteritis | 5 |
| Tuberculosis | 5 |
| Anaemia | 5 |
| Upper respiratory tract infection | 3 |
| Undescended testicles | 3 |
| Nephrotic syndrome | 3 |
| Cyst | 3 |
| Urinary tract infection | 3 |
| Acute viral hepatitis | 3 |
| Pertussis | 2 |
| Bronchitis | 2 |
| Rickets | 2 |
| Hepatitis | 2 |
| Hyperkalaemia | 2 |
| Renal vein thrombosis | 2 |
| Intestinal parasite | 2 |
| Stunted growth | 1 |
| Rickets | 1 |
| Bacterial meningitis | 1 |
| Severe acute malnutrition | 1 |
| Atherosclerosis | 1 |
| Traumatic brain injury | 1 |
| Syncope | 1 |
| Tonsillopharyngitis | 1 |
| Allergic rhinitis | 1 |
| Type 1 diabetes | 1 |
| Acute kidney failure | 1 |
| Acute glomerulonephritis | 1 |
| Rheumatic heart disease | 1 |
| Closed femoral shaft fracture | 1 |
| Recurrent disease | 1 |
| Juvenile rheumatoid arthritis | 1 |
| Dermatitis | 1 |
| Haemangioma | 1 |
| Hemiparesis | 1 |
| Hepatitis-associated aplastic anaemia | 1 |
| Hydrocele | 1 |
| Hypoalbuminemia | 1 |
| Acute febrile illness | 1 |
| Pneumonia | 1 |
| Mesenteric lymphadenitis | 1 |
| Molluscum contagiosum | 1 |
| Myalgia | 1 |
| Bilateral leg deformity | 1 |
| Obstructive sleep apnoea | 1 |
| Childhood stroke | 1 |
| Meningitis | 1 |
| Unclear | 7 |
| No information | 35 |
| Total | 161 |
| *Note.* The total count exceeds 139 because some participants had more than one condition. | |

| Table A.2 | | | | | | | |
| --- | --- | --- | --- | --- | --- | --- | --- |
| *ATEC: item statistics* | | | | | | | |
|  | | Control  (*n* = 139) | Case  (*n* = 139) |  | | Control  (*n* = 139) | Case  (*n* = 139) |
|  | | *Median* (*Q1*–*Q3*) | |  |  | *Median* (*Q1*–*Q3*) | |
| Speech/Language/Communication^1^ | | | | | Sensory/Cognitive Awareness^1^ | | |
| 1. | 0 (0–0) | | 0 (0–1) | 1. | | 0 (0–0) | 0 (0–2) |
| 2. | 0 (0–0) | | 1 (0–2) | 2. | | 0 (0–0) | 1 (0–2) |
| 3. | 0 (0–0) | | 1 (0–2) | 3. | | 0 (0–0) | 0 (0–2) |
| 4. | 0 (0–0) | | 0 (0–2) | 4. | | 0 (0–0) | 0 (0–1) |
| 5. | 0 (0–0) | | 2 (0–2) | 5. | | 0 (0–1) | 2 (1–2) |
| 6. | 0 (0–0) | | 2 (1–2) | 6. | | 0 (0–0) | 1 (0–2) |
| 7. | 0 (0–1) | | 2 (1–2) | 7. | | 0 (0–0) | 1 (0–2) |
| 8. | 0 (0–1) | | 2 (2–2) | 8. | | 0 (0–1) | 2 (1–2) |
| 9. | 0 (0–0) | | 2 (2–2) | 9. | | 0 (0–1) | 2 (1–2) |
| 10. | 0 (0–0) | | 2 (2–2) | 10. | | 0 (0–0.5) | 2 (0–2) |
| 11. | 0 (0–1) | | 2 (2–2) | 11. | | 0 (0–1) | 2 (0–2) |
| 12. | 0 (0–1.5) | | 2 (2–2) | 12. | | 1 (1–2) | 2 (1–2) |
| 13. | 0 (0–1) | | 2 (2–2) | 13. | | 0 (0–0.5) | 1 (0–2) |
| 14. | 0 (0–0) | | 2 (2–2) | 14. | | 0 (0–2) | 2 (0–2) |
| Sociability^2^ | | | | 15. | | 2 (2–2) | 1 (0–2) |
| 1.^†^ | 0 (0–0) | | 1 (0–2) | 16. | | 0 (0–0.5) | 1 (0–2) |
| 2. | 0 (0–1) | | 1 (0–2) | 17. | | 2 (1–2) | 2 (1–2) |
| 3.^†^ | 0 (0–0) | | 1 (0–2) | 18. | | 0 (0–1) | 2 (0–2) |
| 4. | 0 (0–1) | | 1 (1–2) |  | Health/Physical/Behaviour^3^ | | |
| 5.^†^ | 0 (0–0) | | 1 (0–2) | 1. | | 0 (0–1) | 1 (0–3) |
| 6. | 0 (0–0) | | 0 (0–2) | 2. | | 0 (0–0) | 0 (0–3) |
| 7.^†^ | 0 (0–0.5) | | 0 (0–2) | 3. | | 0 (0–0) | 0 (0–3) |
| 8.^†^ | 0 (0–0) | | 1 (0–2) | 4. | | 0 (0–0) | 0 (0–0) |
| 9.^†^ | 0 (0–0) | | 1 (0–2) | 5. | | 0 (0–0) | 0 (0–0) |
| 10.^†^ | 2 (0–2) | | 2 (1–2) | 6. | | 0 (0–0) | 0 (0–1) |
| 11.^†^ | 1 (0–2) | | 0 (0–2) | 7. | | 1 (0–2) | 0 (0–2) |
| 12.^†^ | 0 (0–2) | | 1 (0–2) | 8. | | 0 (0–2) | 0 (0–2) |
| 13.^†^ | 0 (0–0) | | 1 (0–2) | 9. | | 0 (0–1) | 2 (0–3) |
| 14.^†^ | 0 (0–1) | | 2 (1–2) | 10. | | 0 (0–1) | 0 (0–0) |
| 15. | 1 (0–1) | | 1 (1–2) | 11. | | 0 (0–0) | 0 (0–2) |
| 16.^†^ | 0 (0–0) | | 1 (0–2) | 12. | | 0 (0–0) | 0 (0–2) |
| 17.^†^ | 0 (0–0) | | 0 (0–1) | 13. | | 0 (0–0) | 2 (0–2) |
| 18.^†^ | 0 (0–1) | | 2 (0–2) | 14. | | 0 (0–0) | 0 (0–2) |
| 19. | 0 (0–0) | | 1 (0–2) | 15. | | 0 (0–0) | 0 (0–1) |
| 20. | 0 (0–0) | | 0 (0–2) | 16. | | 0 (0–1) | 0 (0–1) |
|  | |  |  | 17. | | 0 (0–0) | 0 (0–0) |
|  |  | |  | 18. | | 0 (0–0) | 0 (0–2) |
|  |  | |  | 19. | | 0 (0–0) | 0 (0–2) |
|  |  | |  | 20. | | 0 (0–0) | 1 (0–2) |
|  |  | |  | 21. | | 0 (0–0) | 0 (0–1) |
|  | |  |  | 22. | | 0 (0–1) | 1 (0–2) |
|  |  | |  | 23. | | 0 (0–1) | 1 (0–2) |
|  |  | |  | 24. | | 0 (0–0) | 1 (0–2) |
|  |  | |  | 25. | | 0 (0–0) | 1 (0–2) |
| *Note.* *Q1*: lower quartile, *Q3*: upper quartile. The Speech/Language/Communication and Sensory/Cognitive Awareness subscales have been reverse-coded.  ^1^0: *Yes*, 1: *Sometimes*, 2: *No*  ^2^0: *No, s/he doesn’t*, 1: *Sometimes*, 2: *Yes, s/he does*  ^3^0: *Not a problem*, 1: *Minor problem*, 2: *Moderate problem*, 3: *Serious problem*  ^†^Items that have been additionally reverse-coded because they indicated a skill rather than a difficulty in the translated version | | | | | | | |

| Table A.3 | | |  | |  |  | | |
| --- | --- | --- | --- | --- | --- | --- | --- | --- |
| *PedsQL™ FIM: item statistics* | | |  | |  |  | | |
|  | Control  (*n* = 138) | Case  (*n* = 139) |  | | Control  (*n* = 138) | Case  (*n* = 139) | | |
|  | *Median* (*Q1*–*Q3*) | |  |  | *Median* (*Q1*–*Q3*) | | | |
| Physical Functioning*^1^* | | |  | Communication*^1^* | |  |  |  |
| 1. | 50 (50–100) | 50 (25–50) | 1. | | 75 (50–100) | 50 (25–75) | | |
| 2. | 75 (50–100) | 50 (25–75) | 2. | | 75 (75–100) | 75 (25–75) | | |
| 3. | 75 (50–100) | 50 (25–75) | 3. | | 100 (75–100) | 100 (75–100) | | |
| 4. | 50 (50–75) | 50 (50–87.5) |  | Worry*^1^* | | |  |  |
| 5. | 75 (50–100) | 50 (50–75) | 1. | | 50 (25–50) | 25 (0–50) | | |
| 6. | 100 (75–100) | 100 (75–100) | 2. | | 50 (25–75) | 25 (0–75) | | |
| Emotional Functioning*^1^* | | | 3. | | 75 (50–100) | 25 (0–75) | | |
| 1. | 50 (25–75) | 25 (0–50) | 4. | | 75 (50–100) | 25 (0–75) | | |
| 2. | 75 (31.25–100) | 25 (12.5–50) | 5. | | 50 (0–100) | 0 (0–25) | | |
| 3. | 75 (50–100) | 50 (25–50) |  | Daily Activities*^1^* | |  |  |  |
| 4. | 75 (50–100) | 50 (25–50) | 1. | | 75 (50–100) | 50 (25–75) | | |
| 5. | 75 (50–100) | 75 (50–100) | 2. | | 75 (50–100) | 50 (25–75) | | |
| Social Functioning*^1^* | | | 3. | | 75 (50–100) | 50 (25–75) | | |
| 1. | 75 (50–100) | 50 (25–100) |  | Family Relationships*^1^* | | | |  |
| 2. | 75 (56.25–100) | 50 (25–75) | 1. | | 75 (75–100) | 75 (50–100) | | |
| 3. | 75 (50–100) | 25 (25–75) | 2. | | 75 (75–100) | 75 (50–100) | | |
| 4. | 75 (50–100) | 50 (25–75) | 3. | | 75 (75–100) | 75 (50–100) | | |
| Cognitive Functioning*^1^* | | | 4. | | 75 (75–100) | 75 (50–100) | | |
| 1. | 75 (56.25–100) | 75 (50–75) | 5. | | 75 (50–100) | 50 (25–100) | | |
| 2. | 75 (56.25–100) | 75 (50–75) |  | |  |  | | |
| 3. | 75 (75–100) | 75 (50–100) |  | |  |  | | |
| 4. | 75 (75–100) | 75 (50–100) |  | |  |  | | |
| 5. | 75 (75–100) | 75 (50–100) |  | |  |  | | |
| *Note.* *Q1*: lower quartile, *Q3*: upper quartile. All items have been reverse-coded and rescaled from 0–4 to 0, 25, 50, 75 and 100.  ^1^0: *Almost always*, 25: *Often*, 50: *Sometimes*, 75: *Almost never*, 100: *Never* | | | | | | | | |
